# Supplementary material for: Pyridylpiperazine-based allosteric inhibitors of RND-type multidrug efflux pumps
Source: Nat Commun. 2022 Jan 10;13:115. doi: 10.1038/s41467-021-27726-2 (PMC8749003; doi:10.1038/s41467-021-27726-2)
Supplement: Supplementary file 1 — Supplementary information [file 41467_2021_27726_MOESM1_ESM.pdf]

Supplementary Information

Title:

Pyridylpiperazine-based allosteric inhibitors of RND-type multidrug efflux pumps

Author list:

Coline Plé[1#], Heng-Keat Tam[2,5#], Anais Vieira Da Cruz[3], Nina Compagne[3], Juan-Carlos Jiménez-Castellanos[1], Reinke T. Müller[2], Elizabeth Pradel[1], Wuen Ee Foong[2], Giuliano Malloci[4], Alexia Ballée[3], Moritz A. Kirchner[2], Parisa Moshfegh[3], Adrien Herledan[3], Andrea Herrmann[2], Benoit Deprez[3], Nicolas Willand[3], Attilio Vittorio Vargiu[4], Klaas M. Pos[2*], Marion Flipo[3*] and Ruben C. Hartkoorn[1*]

[#] Joint first authors: Coline Plé and Heng-Keat Tam: These authors contributed equally

* Joint last and corresponding authors: Klaas M. Pos, Marion Flipo and Ruben C. Hartkoorn: These authors jointly supervised this work

pos@em.uni-frankfurt.de

marion.flipo@univ-lille.fr

ruben.hartkoorn@inserm.fr

Affiliations:

[1] Univ. Lille, CNRS, Inserm, CHU Lille, Institut Pasteur Lille, U1019 - UMR 9017 - CIIL - Center for Infection and Immunity of Lille, F-59000 Lille, France.

[2] Institute of Biochemistry, Goethe-University Frankfurt, Max-von-Laue-Str. 9, D-60438 Frankfurt am Main, Germany.

[3] Univ. Lille, Inserm, Institut Pasteur de Lille, U1177 - Drugs and Molecules for Living Systems, F-59000, Lille, France.

[4] Department of Physics, University of Cagliari, 09042 Monserrato (Cagliari), Italy.

[5] Present address: Hengyang Medical College, University of South China, Hengyang 421002, Hunan Province, China.

Table of content

Table S1: Small molecule screening data……………………………………………………………………………….......**3**
Table S2: MIC of *E. coli* BW25113 in the absence and the presence of the BDM73185 **(1)** as determined by resazurin assay……….**4**
Table S3: MIC of *E. coli* BW25113 and *acrAB-tolC* mutants in the absence and the presence of BDM73185 **(1)** as determined by resazurin assay…………………………………………………………………………….**4**
Table S4: Characteristics and MIC of *E. coli* BW25113 and BDM73185 **(1)** resistant mutants…………….**5**
Table S5: Pyridomycin boosting activity of EPIs on the isolated *E. coli* BW25113 BDM73185 **(1)** resistant mutants……..**5**
Table S6: Validation of genetic causality of isolated BDM73185 resistance mutations by reverse genetics……….**6**
Table S7: Summary of BDM88855.HCl (**9'**) (100 µM) mediated boosting of antibiotics in *E. coli* BW25113, *E. coli* BW25113 *ΔacrAB* and the two isolated pyridylpiperazine resistant mutants carrying the S450P and A446P AcrB substitutions…………………………………………………………………………………**7**
Table S8: Data collection and refinement statistics……………………………………………………………………**8**
Table S9: Superimposition of individual L, T, or O protomers from inhibitor bound AcrB structures with minocycline bound wildtype AcrB (PDB: 4DX5) or R971A variant (PDB: 4U96) with or without bound inhibitor………**9**
Table S10: Validation of BDM88855 (9) binding pocket by targeted mutagenesis of *acrB*………………..**10**
Table S11: Local correlation coefficients (CC) and CC_{peak} as calculated by phenix.polder………………….**11**
Table S12: Primer sequences for site directed mutagenesis of *acrB* (with 5' phosphorylated)…………**11**
Fig S1: Effect of BDM88855.HCl (**9'**) on berberine accumulation of *E. coli* BW25113 Δ*acrB* producing different AcrB variants…….**12**
Fig. S2: Thermal shift assays (TSAs) of AcrB wildtype (WT) and the A446P variant in dependence of the BDM88855.HCl (9') concentration………………………………………………………………………………………..**13**
Fig. S3: Pyridylpiperazine inhibitor binding pocket comprising the TM4, TM5 and TM10 interacting side chains in the TM domain of the AcrB L protomer…………………………………………………………………….**14**
Fig. S4: Superimposition of the D407/D408/K940 proton relay site of the apo- and BDM88855 (**9**)-bound structure……..**15**
Fig. S5: Configuration of the proton-binding site in AcrB in the absence or presence of BDM88855..**15**
Fig. S6: Drug susceptibility profiles of *E. coli* BW25113 Δ*acrB* producing single-alanine substitution AcrB variants……**16**
Fig. S7: Drug susceptibility profiles of *E. coli* BW25113 Δ*acrB* producing functional AcrB variants in the absence and presence of BDM88855 **(9)**……………………………………………………………………………………**16**
Fig S8: Western blot analysis of equal amounts of *E. coli* cell extracts producing the indicated AcrB protein…….**17**
Fig. S9: Model system setup to investigate the putative uptake route of BDM88832 (**8**) towards its experimental binding site in the TM region of the L monomer of AcrB……………………………………………**18**
Fig. S10: Accumulation of BDM88832 (**8**) at the cytoplasmic entrance of the TM domain of the L protomer of AcrB. ……….**19**
Fig. S11: Synthesis scheme of compounds **2-4**…………………………………………………………………………**21**
Fig. S12: Synthesis scheme of compounds **5** and **6**……………………………………………………………………**22**
Fig. S13: Synthesis scheme of compounds **7** and **8**. ………………………………………………………………….**23**
Fig. S14: Synthesis scheme of compounds **9'** and **10**…………………………………………………………………**24**
Fig. S15: Synthesis scheme of compounds **11** and **12**………………………………………………………………**25**

Table S1: Small molecule screening data

Category	Parameter	Description
Assay	Type of assay	Whole organism viability assay (phenotypic)
	Target	*E. coli* viability
	Primary measurement	Bacterial reduction of resazurin to resorufin by fluorescence (Ex: 530 nm Em: 590 nm) measured using a POLARstar Omega, (BMG Labtech)
	Key reagents	Resazurin sodium salt (Alfa Aesar)
	Assay protocol	For the screening of potential EPIs, a chemical library of 1280 compounds (at 100 mM in DMSO) were transferred (in duplicate) by acoustic technology (Echo® 550, Labcyte Inc) to a destination 384-well plate (150 nL/well). *Escherichia coli* BW25113 was then thawed from frozen stocks and diluted to an OD_{600} of 0.0004 in cation-adjusted Mueller-Hinton broth (CAMHB). This culture was split in two, and spiked with either DMSO (control for booster activity alone), or pyridomycin (final concentration of 5 µg/mL), and transferred to the destination plates containing the chemical library (50 µL per well). Following culture (5h, 37°C), *E. coli* viability was evaluated using the resazurin reduction assay and measured by fluorescence (POLARstar Omega, BMG Labtech: Ex: 530 nm Em: 590 nm).
	Additional comments	N/A
Library	Library size	1280
	Library composition	Fragments
	Source	Commercially available molecules and unique in-house synthesized compounds
	Additional comments	Compounds are stored at -20°C in 100 mM DMSO stock solution
Screen	Format	384-well black plates with transparent bottom (Greiner Bio-One)
	Concentration(s) tested	300 µM final test compound concentration,<br>0.3% DMSO<br>Bacteria grown in the absence or presence of 5 µg/mL pyridomycin
	Plate controls	In column 1, 2, 23 and 24 alternating wells contained either 0.2 µg/mL ciprofloxacin (positive control) or 0.3% DMSO (negative control)
	Reagent/ compound dispensing system	Viafill (Integra) for dispensing bacteria
	Detection instrument and software	N/A
	Assay validation/QC	On-plate Z-factor was > 0.8.
	Correction factors	N/A
	Normalization	N/A
	Additional comments	Test compounds were added to 384-well plates by acoustic technology (Echo® 550, Labcyte Inc).<br>Pyridomycin was added to bacteria prior to dispensing them into the 384-well plates.
Post-HTS analysis	Hit criteria	> 80% inhibition of resazurin turnover by compounds in the presence of 5 µg/mL pyridomycin AND < 20% inhibition of resazurin turnover by compounds in the absence of 5 µg/mL pyridomycin
	Hit rate	1 hit / 1280 compounds tested
	Additional assay(s)	The activity of hits was confirmed by repeating the experiments in 96-well format using a new batch of the hit compound.
	Confirmation of hit purity and structure	BDM73185 (**1**) was purchased from Maybridge, structure and purity were verified analytically (see suppl. chemistry).
	Additional comments	N/A

Table S2: MIC of *E. coli* BW25113 in the absence and the presence of the BDM73185 **(1)** as determined by resazurin assay. Data are from a single experiment. Source data are provided as a Source Data file.

	**MIC95 (µg/mL) without boosting compound**	**MIC95 (µg/mL) with 300 µM BDM73185 (1) (fold shift)**
Chloramphenicol	10	1.25 (8-fold)
Pyridomycin	25	6.25 (4-fold)
Ciprofloxacin	0.0062	0.0031 (2-fold)
Tetracycline	0.625	0.156 (4-fold)
Erythromycin	6.25	3.125 (2-fold)
Ampicillin	6.25	3.125 (2-fold)
Rifampicin	3.125	3.125 (1-fold)
Streptomycin	0.78	1.56 (0.5-fold)
Kanamycin	0.78	1.56 (0.5-fold)

Table S3: MIC of *E. coli* BW25113 and *acrAB-tolC* mutants in the absence and the presence of BDM73185 **(1)** as determined by resazurin assay. Data are from a single experiment. Source data are provided as a Source Data file.

**Strains**	**BDM73185 (1) (µM)**	**MIC95 (µg/mL) of Pyridomycin**	**MIC95 (µg/mL) of Chloramphenicol**	**MIC95 (µg/mL) of Streptomycin**
E. coli BW25113	0	25	5.0	2.5
	150	12.5	1.25	2.5
	300	6.25	0.625	2.5
E. coli BW25113 *ΔacrA*	0	0.5	0.625	1.25
	150	0.5	0.625	2.5
	300	0.5	0.625	2.5
E. coli BW25113 *ΔacrB*	0	0.5	0.625	2.5
	150	0.5	0.625	5
	300	0.5	0.625	5
E. coli BW25113 *ΔtolC*	0	1.0	0.625	2.5
	150	0.5	0.625	5
	300	0.5	0.625	5

Table S4: Characteristics and MIC of *E. coli* BW25113 and BDM73185 **(1)** resistant mutants. Data are from a single experiment. Source data are provided as a Source Data file.

Strains	Isolate selected on:	*acrB* genotype	Pyridomycin MIC95 (µg/mL) without and [with 300 µM BDM73185 (1)]	Erythromycin MIC95 (µg/mL) without and [with 300 µM BDM73185 (1)]
E. coli BW25113		wt	25 [6.25]	25 [12.5]
E. coli BW25113 *RC 1.25_1*	1.25 µg/mL erythromycin, 600 µM BDM73185 **(1)**	t1348c (S450P)	25 [25]	25 [25]
E. coli BW25113 *RC 1.25_2*	1.25 µg/mL erythromycin, 600 µM BDM73185 **(1)**	g1336c (A446P)	50 [25]	50 [25]
E. coli BW25113 *RC 2.5_1*	2.5 µg/mL erythromycin, 600 µM BDM73185 **(1)**	t1348c (S450P)	25 [25]	50 [50]
E. coli BW25113 *RC 5_1*	5 µg/mL erythromycin, 600 µM BDM73185 **(1)**	t1348c (S450P)	50 [50]	50 [50]

Table S5: Pyridomycin boosting activity of EPIs on the isolated *E. coli* BW25113 BDM73185 **(1)** resistant mutants. Data are from a single experiment with two technical replicates. Source data are provided as a Source Data file.

Strains	*acrB* genotype	Effective EPI concentration to prevent *E. coli* BW25113 growth in the presence of 8 µg/mL Pyridomycin		
		BDM73185 (1) (µM)	NMP (µM)	PAβN (µM)
E. coli BW25113	wt	62.5 - 125	500	15.6
E. coli BW25113 *RC 1.25_1*	t1348c (S450P)	1000 - 2000	500	15.6
E. coli BW25113 *RC 1.25_2*	g1336c (A446P)	5000- 1000	250	15.6
E. coli BW25113 *RC 2.5_1*	t1348c (S450P)	1000 - 2000	500	15.6
E. coli BW25113 *RC 5_1*	t1348c (S450P)	1000	500	15.6

Table S6: Validation of genetic causality of isolated BDM73185 resistance mutations by reverse genetics. Table shows the antibiotic susceptibility of the isolated *E. coli* BW25113 BDM73185 (**1**) resistant mutants, and the genetically engineered *E. coli* BW25113 *ΔacrB*, complemented with wildtype or mutated *acrB* using Red recombineering. Antibiotic susceptibility is shown in the absence and presence of BDM73185 (**1**) (300 µM) or BDM88855.HCl (**9'**) (100 µM), and clearly shows that both the isolated and engineered isolates have the same phenotype. Data are mean MIC (range) of at least 3 independent replicates. Source data are provided as a Source Data file.

**Strains**	***acrB* genotype**	**Erythromycin MIC (µg/mL)**		
		**Alone**	**With 300 µM BDM73185 (1)**	**With 100 µM BDM88855.HCl (9')**
E. coli BW25113	wt	25	10.4 (6.25-12.5)	6.25
BW25113_*RC 1.25-1 (AcrB:S450P)*	t1348c (S450P)	25	33.3 (25-50)	25
BW25113_*RC 1.25-2 (AcrB:A446P)*	g1336c (A446P)	50	33.3 (25-50)	29.2 (12.5-50)
BW25113_*ΔacrB*	*ΔacrB*	3.13	3.13	4.17 (3.13-6.25)
BW25113_*ΔacrB::acrB(WT)*	wt	37.5 (25-50)	10.4 (6.25-12.5)	6.25
BW25113_*ΔacrB::acrB(S450P)*	t1348c (S450P)	18.75 (12.5-25)	33.3 (25-50)	33.3 (25-50)
BW25113_*ΔacrB::acrB(A446P)*	g1336c (A446P)	25	25	25

**Strains**	***acrB* genotype**	**Pyridomycin MIC (µg/mL)**		
		**Alone**	**With 300 µM BDM73185 (1)**	**With 100 µM B BDM88855.HCl (9')**
E. coli BW25113	wt	10.9 (6.25-12.5)	2.08 (1.56-3.13)	0.65 (0.39-0.78)
BW25113_*RC 1.25-1 (AcrB:S450P)*	t1348c (S450P)	10.9 (6.25-12.5)	10.4 (6.25-12.5)	10.4 (6.25-12.5)
BW25113_*RC 1.25-2 (AcrB:A446P)*	g1336c (A446P)	12.5	8.33 (6.25-12.5)	12.5
BW25113_*ΔacrB*	*ΔacrB*	0.2	0.2	0.2
BW25113_*ΔacrB::acrB(WT)*	wt	10.9 (6.25-12.5)	2.08 (1.56-3.13)	0.52 (0.39-0.78)
BW25113_*ΔacrB::acrB(S450P)*	t1348c (S450P)	12.5	12.5	12.5
BW25113_*ΔacrB::acrB(A446P)*	g1336c (A446P)	12.5	6.25	12.5

**Strains**	***acrB* genotype**	**Oxacillin MIC (µg/mL)**		
		**Alone**	**With 300 µM BDM73185 (1)**	**With 100 µM BDM88855.HCl (9')**
E. coli BW25113	wt	>200	25	4.17 (3.13-6.25)
BW25113_*RC 1.25-1 (AcrB:S450P)*	t1348c (S450P)	>200	200	>200 (200->200)
BW25113_*RC 1.25-2 (AcrB:A446P)*	g1336c (A446P)	>200	200	200
BW25113_*ΔacrB*	*ΔacrB*	3.13	1.82 (0.78-3.13)	1.04 (0.78-1.56)
BW25113_*ΔacrB::acrB(WT)*	wt	>200	100 (50-200)	7.29 (3.13-12.5)
BW25113_*ΔacrB::acrB(S450P)*	t1348c (S450P)	>200	>200	>200
BW25113_*ΔacrB::acrB(A446P)*	g1336c (A446P)	>200	200	200

Table S7: Summary of BDM88855.HCl (**9'**) (100 µM) mediated boosting of antibiotics in *E. coli* BW25113, *E. coli* BW25113 *ΔacrAB* and the two isolated pyridylpiperazine resistant mutants carrying the S450P and A446P AcrB substitutions. Data are the mean MIC_{95} values (µg/mL) ± SEM of at least 2 independent biological replicates (no SEM is given when all replicates gave the same MIC) as measured using the resazurin reduction assay to determine bacterial viability. Source data are provided as a Source Data file.

***E. coli* strain →**	**BW25113**			**Resistant clone 1.25_1 (S450P)**			**Resistant clone 1.25_2 (A446P)**			***ΔacrAB***
**100 µM BDM88855.HCl (9') →**	**Without**	**With**	**Fold BOOST**	**Without**	**With**	**Fold BOOST**	**Without**	**With**	**Fold BOOST**	**Without**
**ANTIBIOTIC MOLECULES**										
Oxacillin	>100	3.0	>33.3	>100	>100	1.0	>100	>100	1.0	1.2 ± 0.37
Linezolid	100	3.1 ± 0.07	32.6	100.00	31 ± 9.4	3.2	67 ± 22	18.5 ± 6.5	3.6	4.8 ± 2.3
Novobiocin	>100	12.0	>8.3	>100	>100	1.0	>100	>100	1.0	3.8 ± 1.0
Fusidic Acid	>100	12.0	>8.3	>100	>100	1.0	>100	>100	1.0	10. ± 7.0
Pyridomycin	9 ± 3	0.75 ± 0.25	12.0	12	12	1.0	12	12	1.0	0.62 ± 0.19
Chloramphenicol	4.5 ± 1.5	0.5	9.0	3.0	2.0	1.5	2.0	2.0	1.0	0.86 ± 0.56
Erythromycin	18.5 ± 6.5	4.0 ± 2.0	4.6	18.5 ± 6.5	18.5 ± 6.5	1.0	38 ± 13	25	1.5	5.6 ± 1.0
Ciprofloxacin	0.052 ± 0.014	0.013 ± 0.004	4.0	0.063 ± 0.00022	0.063 ± 0.00018	1.0	0.063 ± 0.00022	0.063 ± 0.00022	1.0	0.012 ± 0.0047
Piperacillin	0.39	0.10	3.9	0.39	0.39	1.0	0.30 ± 0.10	0.20	1.5	0.24 ± 0.22
Tetracycline	0.75 ± 0.25	0.25	3.0	0.50	0.50	1.0	0.50	0.50	1.0	0.42 ± 0.18
Triclosan	0.045 ± 0.005	0.026 ± 0.018	1.7	0.039 ± 0.0069	0.029 ± 0.011	1.3	0.045 ± 0.005	0.033 ± 0.008	1.4	0.011 ± 0.0023
Ampicillin	4.5 ± 1.5	2.5 ± 0.5	1.8	6.0	6.0	1.0	2.5 ± 0.5	4.5 ± 1.5	0.6	3.5 ± 1.4
Ceftazidime	0.12	0.25	0.5	0.25	0.25	1.0	0.19 ± 0.07	0.25	0.7	0.35 ± 0.18
Streptomycin	3.13 ± 0.005	6.3	0.5	4.7 ± 1.6	9.4 ± 3.1	0.5	4.7 ± 1.6	9.4 ± 3.1	0.5	2.8 ± 0.50
Aztreonam	0.060	0.09 ± 0.03	0.7	0.060	0.12	0.5	0.060	0.12	0.5	0.12 ± 0.032
Gentamicin	0.59 ± 0.2	1.60	0.4	0.98 ± 0.59	2.0 ± 1.2	0.5	0.59 ± 0.20	1.2 ± 0.39	0.5	0.43 ± 0.14
Kanamycin	2.34 ± 0.78	6.3	0.4	3.9 ± 2.3	9.4 ± 3.1	0.4	2.3 ± 0.78	4.7 ± 1.6	0.5	ND
TPPT	>100	12.0	>8.3	>100	>100	1.0	>100	>100	1.0	ND
Tigecycline	0.25	0.12	2.1	0.25	0.25	1.0	0.25	0.19 ± 0.07	1.4	ND
Rifampicin	9 ± 3	9 ± 3	1.0	9 ± 3	9 ± 3	1.0	12	12	1.0	ND
Vancomycin	100.0	>100	1.0	100	<100	1.0	~100	<100	1.0	ND
Rifabutin	9 ± 3	9 ± 3	1.0	9 ± 3	9 ± 3	1.0	12	12	1.0	ND
Meropenem	0.25	0.25	1.0	0.25	0.38 ± 0.13	0.7	0.25	0.25	1.0	ND

ND = Not determined

Table S8: Data collection and refinement statistics.

	AcrB + BDM88855 (9)	AcrB-R971A + BDM88832 (8)	AcrB-R971A + BDM88855 (9)
**PDB code**	**7OUK**	**7OUL**	**7OUM**
**Data collection**			
Beamline	PX2A, SOLEIL Synchrotron	PX2A, SOLEIL Synchrotron	PX1, SOLEIL Synchrotron
PDB accession			
Space group:	$P2_12_12_1$	$P2_12_12_1$	$P2_12_12_1$
Cell constants a; b; c (Å)	146.13, 159.65, 245.50	146.41, 160.01, 245.48	146.21, 161.63, 244.44
α; β; γ (°)	90.0, 90.0, 90.0	90.0, 90.0, 90.0	90.0, 90.0, 90.0
Resolution	49.10–2.60 (2.64–2.60)	49.43–2.80 (2.85–2.80)	49.51– 2.45 (2.49-2.45)
Unique reflections	176116 (8611)	142096 (6938)	212143 (10384)
Completeness overall (%)	99.9 (99.7)	100.0 (100.0)	100.0 (100.0)
Multiplicity (%)	13.9 (14.0)	13.9 (14.3)	13.9 (14.0)
$\langle I\rangle/\langle\sigma(I)\rangle$	9.5 (1.7)	9.3 (1.6)	6.5 (1.5)
R_{meas}	0.235 (2.158)	0.285 (2.134)	0.292 (2.127)
R_{merge}	0.226 (2.081)	0.275 (2.058)	0.281 (2.050)
R_{pim}	0.063 (0.572)	0.076 (0.562)	0.078 (0.565)
CC(1/2)	0.998 (0.597)	0.998 (0.603)	0.972 (0.405)
**Refinement**			
No. of reflections	167178	135011	201567
R_{work} / R_{free}	0.2365 / 0.2625	0.2422 / 0.2611	0.2280 / 0.2556
**No. atoms**			
Protein / Ligand + ion / Water	25971 / 599 / 365	25898 / 365 / 173	25944 / 519 / 671
**B-factors**			
Protein / Ligand + ion / Water	59.59 / 71.74 / 39.92	61.84 / 73.05 / 34.89	53.08 / 63.73 / 38.03
**r.m.s. deviations from ideal values**			
Bond length (Å)	0.0016	0.0015	0.0016
Bond angles (º)	1.1717	1.1721	1.1689
**Φ, Ψ angle distribution for residues**			
In allowed regions / In disallowed regions (%)	100.0 / 0.0	99.9 / 0.1	99.9 / 0.1

Table S9: Superimposition of individual L, T, or O protomers from inhibitor bound AcrB structures with minocycline bound wildtype AcrB (PDB: 4DX5) or R971A variant (PDB: 4U96) with or without bound inhibitor. As a control, fusidic acid bound AcrB (PDB: 5JMN) is compared with minocycline bound wildtype AcrB. The numbers indicate the root mean square deviation (RMSD, in Å) between the C_α atoms of each superimposed structure. A larger number indicates a larger deviation between the structures. The values indicating the largest deviations are indicated in bold.

**AcrB structures**	**Minocycline bound AcrB (RMSD of C_α)**		
	**L**	**T**	**O**
Fusidic acid bound AcrB	0.32 Å	0.47 Å	0.22 Å
R971A	**0.80 Å**	**0.90 Å**	0.26 Å
BDM88855 (**9**) bound to TM domain of wildtype AcrB	**0.89 Å**	0.52 Å	0.26 Å
BDM88855 (**9**) bound to TM domain of AcrB-R971A	**1.02 Å**	0.55 Å	0.27 Å
BDM88832 (**8**) bound to TM domain of AcrB-R971A	**1.05 Å**	0.62 Å	0.31 Å

**AcrB structures**	**AcrB-R971A (RMSD of C_α)**		
	**L**	**T**	**O**
Fusidic acid bound AcrB	**0.83 Å**	**0.84 Å**	0.30 Å
R971A	**-**	**-**	-
BDM88855 (**9**) bound to TM domain of wildtype AcrB	0.52 Å	**0.73 Å**	0.32 Å
BDM88855 (**9**) bound to TM domain of AcrB-R971A	0.51 Å	0.64 Å	0.25 Å
BDM88832 (**8**) bound to TM domain of AcrB-R971A	0.54 Å	0.60 Å	0.27 Å

Table S10: Validation of BDM88855 (9) binding pocket by targeted mutagenesis of *acrB*. Here the drug susceptibility profiles of *E. coli* BW25113 Δ*acrB* harbouring single-alanine substitution targeting 8 AcrB residues were determined in the absence and presence of BDM88855(**9**). The chosen AcrB amino acid substitutions are located in the inhibitor binding site identified by the X-ray crystal structures. The **upper portion** on the table evaluates the efflux capacity of the recombinant AcrB variants by evaluating their growth rate in the presence of various antibiotics. Data represents the growth rate (in percentage) of *E. coli* producing the AcrB variants, relative to the growth rate of cells harbouring WT AcrB (considered 100% growth) or the catalytically inactive D407N AcrB variant (considered 0% growth). Data show 4 AcrB variants with a significantly diminished growth rate, suggesting that the corresponding mutations prevented proper antibiotic efflux. In the **bottom portion** of the table the susceptibility to BDM88855 (**9**) of the 4 functional AcrB variants was determined. Data are mean ± s.e.m. of *N* = 3–4 biologically independent cells. (The images of all plate dilutions on basis the calculations were done are provided as "**source data**"). Data shows that while WT and the F948A AcrB variant were inhibited by BDM88855 (**9**) the V411A, L442A and E947A AcrB variants were not. Statistics performed by two-sided Student's t-test (* p<0.05 (salmon), ** p<0.005 (red)) relative to AcrB WT (top data) or relative to no inhibitor variant controls (bottom data). Statistics performed by two-sided Student's *t*-test (§ p = <0.005) relative to AcrB WT in the presence of inhibitor (bottom data). Exact p values are available in accompanying source data table. All AcrB substitution variants (except for L944A variant) were well expressed as determined by Western blot analysis (**see Fig. S8**). Source data are provided as a Source Data file.

**BW25113 Δ*acrB* producing recombinant protein**	**Relative bacterial growth rate in the presence of:**				
	**Dicloxacillin**	**Erythromycin**	**Fusidic acid**	**Linezolid**	**TPP**
AcrB (wt) [positive ctl]	100	100	100	100	100
AcrB (D407N) [negative ctl]	0	0	0	0	0
AcrB (V411A)	98 ± 4	99 ± 5	93 ± 4	99 ± 5	96 ± 4
AcrB (I438A)	62 ± 2 **	105 ± 5	68 ± 8 **	65 ± 7 **	97 ± 3
AcrB (L442A)	100 ± 4	104 ± 3	95 ± 5	76 ± 4 **	96 ± 3
AcrB (I445A)	19 ± 8 **	48 ± 17 **	48 ± 7 **	42 ± 6 **	27 ± 7 **
AcrB (I943A)	43 ± 6 **	53 ± 21	77 ± 7 *	23 ± 1 **	60 ± 9 **
AcrB (L944A)	0 ± 0 **	14 ± 5 **	24 ± 4 **	37 ± 6 **	20 ± 6 **
AcrB (E947A)	101 ± 4	105 ± 3	109 ± 5	110 ± 6	94 ± 4
AcrB (F948A)	100 ± 0	100 ± 0	61 ± 11 *	79 ± 13	100 ± 0
AcrB (wt) + BDM88855(**9**)	30 ± 3 **	33 ± 11 **	24 ± 6 **	40 ± 4 **	50 ± 5 **
AcrB (V411A) + BDM88855(**9**)	105 ± 5 §	110 ± 6 §	87 ± 12 §	84 ± 8 §	100 ± 0 §
AcrB (L442A) + BDM88855(**9**)	101 ± 8 §	110 ± 6 §	80 ± 10 §	55 ± 6 **	96 ± 4 §
AcrB (E947A) + BDM88855(**9**)	95 ± 5 §	95 ± 5 §	80 ± 15 §	76 ± 10 * §	100 ± 0 §
AcrB (F948A) + BDM88855(**9**)	0 ± 0 ** §	0 ± 0 ** §	0 ± 0 ** §	17 ± 0 ** §	13 ± 4 ** §

Table S11: Local correlation coefficients (CC) and CC_{peak} as calculated by phenix.polder[1]. Minocycline bound to deep binding pocket (PDB: 4DX5) is shown as reference of the substrate binding. Maps m1, m2, and m3 are defined by Liebschner et al.[11] as map 1 (m1), calculated F_{obs} (= |Fmodel|) assuming that the omitted atoms are present; map 2 (m2), calculated F_{obs} (= |Fmodel|) assuming that the omitted atoms are not present; map 1 and map 2 are synthetic data; map 3 (m3), is a polder map using experimental F_{obs}. The high cross-correlation between the model map m1 (assuming the ligand is present) and the experimentally observed map (m3) clearly indicates the presence of ligand.

	**m1-m2**		**m1-m3**		**m2-m3**	
	CC	CC_{peak}	CC	CC_{peak}	CC	CC_{peak}
Minocycline bound to AcrB (4DX5)	0.55	0.60	**0.89**	**0.85**	0.59	0.62
BDM88855 **(9)** bound to TM domain of AcrB L protomer	0.57	0.61	**0.86**	**0.86**	0.62	0.64
BDM88855 **(9)** bound to funnel domain of AcrB O protomer	0.59	0.59	**0.84**	**0.84**	0.71	0.69
BDM88832 (**8**) bound to TM domain of AcrB-R971A L protomer	0.56	0.70	**0.92**	**0.91**	0.66	0.71
BDM88855 **(9)** bound to TM domain of AcrB-R971A L protomer	0.61	0.66	**0.94**	**0.93**	0.52	0.56
BDM88855 **(9)** bound to funnel domain of AcrB-R971A O protomer	0.57	0.58	**0.86**	**0.87**	0.68	0.64

Table S12: Primer sequences for site directed mutagenesis of *acrB* (with 5’ phosphorylated).

**Plasmid**	**Primer sequences**
pET24acrB$_{His}$	5’-GGATCCCATATGCCTAATTTCTTTATCGATC-3’<br>5’-AAGCTTCTCGAGATGATGATCGACAGTATGGCTG-3’
pET24acrB-V411A$_{His}$	5‘-GCAGTGGTAGAAAACGTTGAGCG-3’<br>5‘-GATGGCGTCATCCACCAACAG-3’
pET24acrB-I438A$_{His}$	5‘-CACAGGGCGCTCTGGTCGG-3’<br>5‘-CCTGCCCCATCGACTTACG-3’
pET24acrB-L442A$_{His}$	5‘-GCGGTCGGTATCGCGATGGT-3’<br>5‘-AGCGCCCTGAATCTGCCCCA-3’
pET24acrB-I445A$_{His}$	5‘-GCCGCGATGGTACTGTCGGC-3’<br>5‘-ACCGACCAGAGCGCCCTGA-3’
pET24acrB-I943A$_{His}$	5‘-GCACTTATCGTCGAATTCGCCAA-3’<br>5‘-CGCGTTCTTCGCCGACAAC-3’
pET24acrB-L944A$_{His}$	5‘-ATCGCCATCGTCGAATTCGCCAA-3’<br>5‘-CGCGTTCTTCGCCGACAAC-3’
pET24acrB-E947A$_{His}$	5‘-GCATTCGCCAAAGACTTGATG-3’<br>5‘-GACGATAAGGATCGCGTTCT-3’
pET24acrB-F948A$_{His}$	5‘-CCGCCAAAGACTTGATGGATAAAG-3’<br>5‘-CTTCGACGATAAGGATCGCGTTC-3’

Supplementary Figures

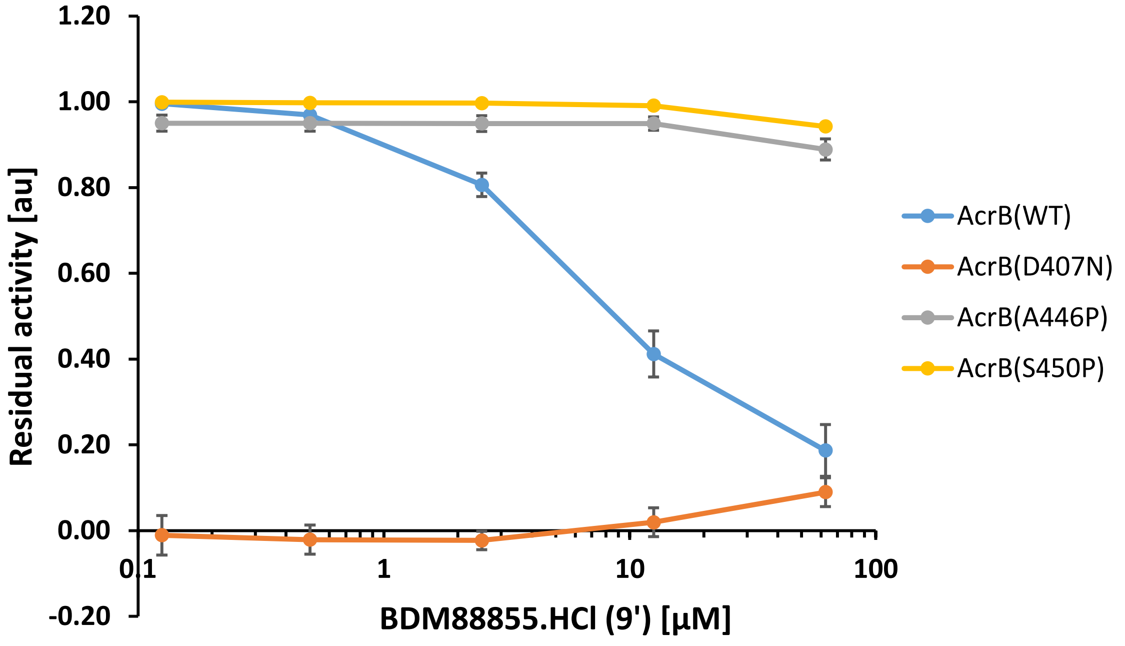

Fig S1: Effect of BDM88855.HCl (**9'**) on berberine accumulation of *E. coli* BW25113 Δ*acrB* producing different AcrB variants. Steady state berberine accumulation fluorescence values of variants in the presence of 0.125 - 62.5 µM BDM88855.HCl (9') were normalized on the difference in fluorescent signal between the wildtype (WT, positive control, 100% activity) and the proton translocation deficient D407N variant (negative control, 0% activity) in the absence of the inhibitor as described in Supplementary Material and Methods. The WT berberine pumping activity is affected by the presence of BDM88855.HCl (9') in a concentration-dependent manner. The escape variants A446P and S450P are *de facto* unaffected by the inhibitor at all concentrations tested. The graph represents the data in a semi-logarithmic manner of the averaged values from six (n=6) measurements of biological replicates. Data are presented as mean values +/- SD. Source data are provided as a Source Data file.

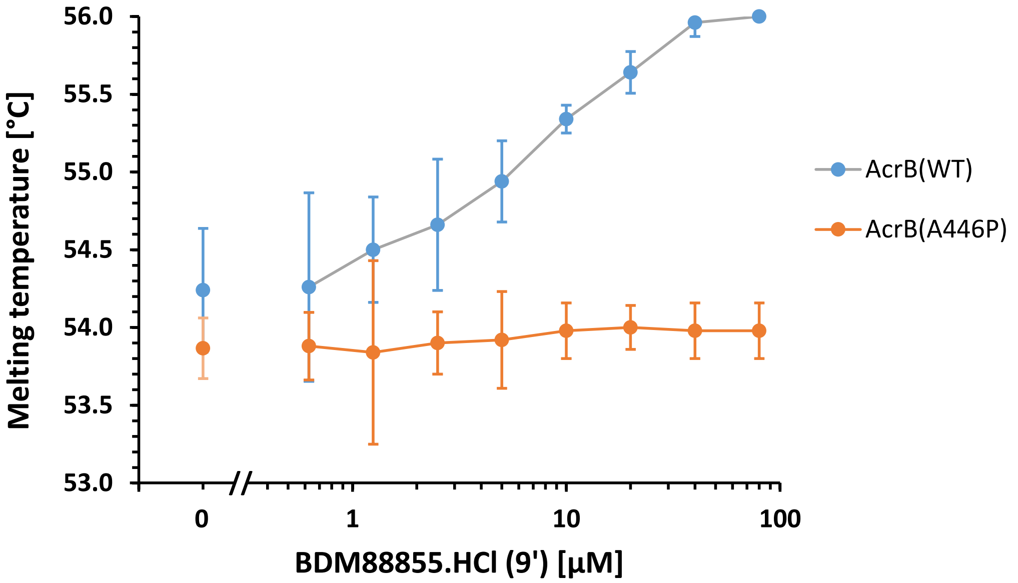

Fig. S2: Thermal shift assays (TSAs) of AcrB wildtype (WT) and the A446P variant in dependence of the BDM88855.HCl (9') concentration. Purified AcrB wildtype or variant were incubated in presence of 0.625 – 80 µM BDM88855.HCl (9') during cysteine-reactive fluorophore N-[4-(7-diethylamino-4-methyl-3-coumarinyl)phenyl]maleimide (CPM)-based melting temperature determination as described in supplementary Material and Methods. Melting temperatures of the WT and the A446P variant are the mean of five (n=5) independent TSA measurements and plotted against the BDM88855.HCl (9') inhibitor concentration in a semi-logarithmic representation. The results show that the stability of the A446P variant remains unaffected in presence of BDM88855.HCl (9') at every concentration tested. In contrast, the melting temperatures of WT AcrB increase in presence of increasing concentration of BDM88855.HCl (9') to a maximum shift of 1.8 ± 0.4°C compared to the melting temperature in absence of BDM88855.HCl (9'). These results indicate that the inhibitor binds to wildtype AcrB and results in stabilization of the protein/inhibitor complex, but does not recognize the A446P escape variant. Data are presented as mean values +/- SD. Source data are provided as a Source Data file.

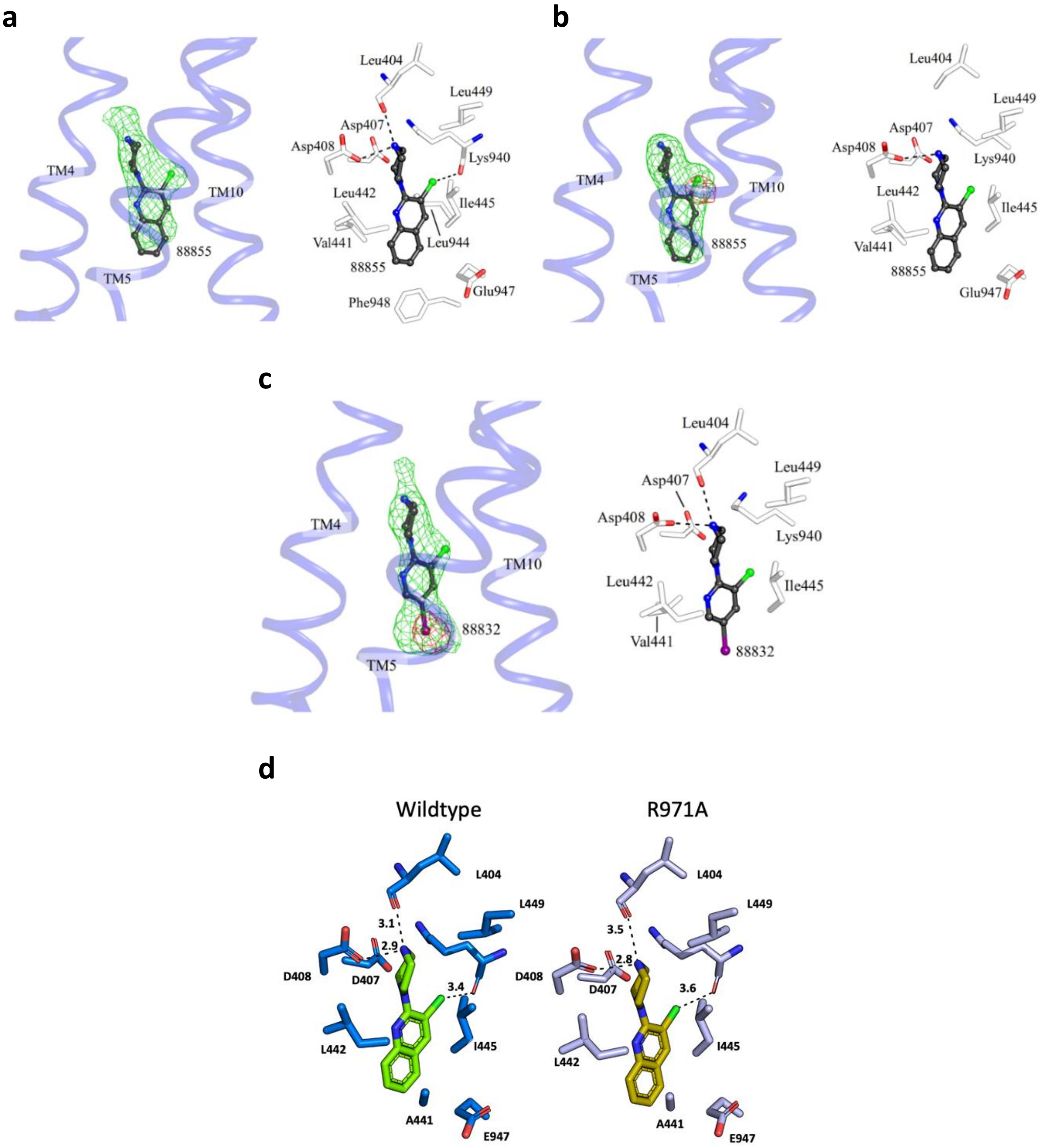

Fig. S3: Pyridylpiperazine inhibitor binding pocket comprising the TM4, TM5 and TM10 interacting side chains in the TM domain of the AcrB L protomer. **a,** BDM88855 (**9**) interaction with side chains from the L protomer TM domain of AcrB; **b,** BDM88855 (**9**) interaction with the side chains of the AcrB-R971A variant L protomer TM domain; **c,** BDM88832 (**8**) interaction with the side chains of the AcrB-R971A variant L protomer TM domain. Polder electron density maps (green mesh, 5 σ) are shown (for values on the polder maps see **Table S11**). Anomalous densities for the bromine atom (bromine moiety of **compound 11**, **b**) and the iodine atom (iodine moiety of BDM88832 (**8**), **c**) are shown as red mesh, contoured at 5.5 and 7.0 σ, respectively**.** Hydrogen and halogen bonds are shown as dashed black lines. BDM88832 (**8**) and BDM88855 (**9**) are depicted as ball and stick models (carbon = black; nitrogen = blue; chlorine = green, iodine = purple). **d,** Structural comparison between BDM88855 (**9**) interaction with side chains from the L protomer TM domain of AcrB (as shown in **a**) and with the side chains of the AcrB-R971A variant L protomer TM domain as shown in **b**. Hydrogen and halogen bonds are shown as dashed black lines. The numbers indicate the distances in Å. BDM88855 (**9**) is depicted as stick model (carbon = green (wildtype) or olive (R971A variant).

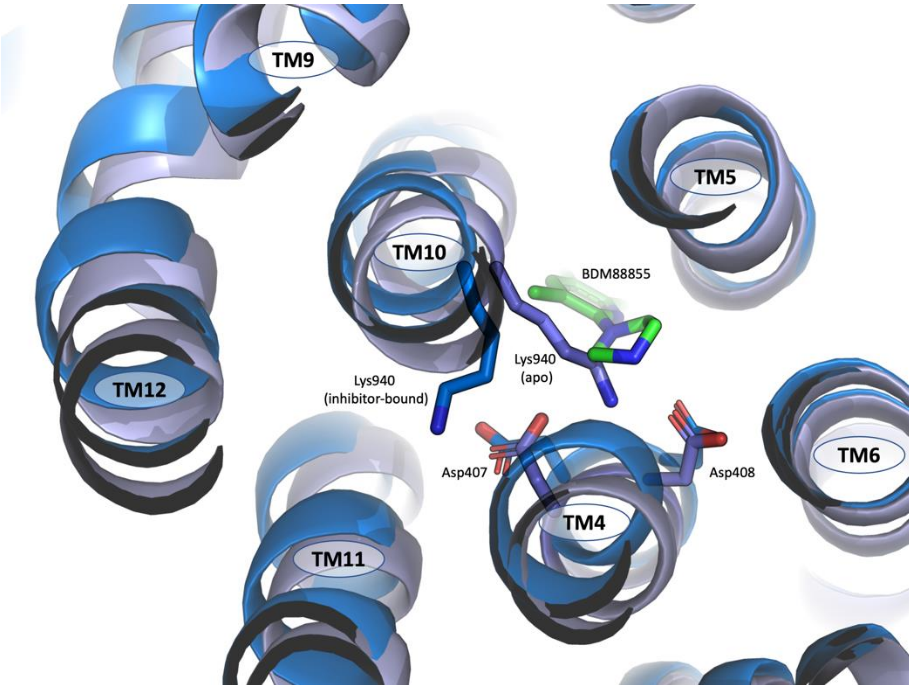

Fig. S4: Superimposition of the D407/D408/K940 proton relay site of the apo- and BDM88855 (**9**) -bound structure. View from the periplasmic side. In this view, Lys940 on TM10 from the apo structure (helices shown as cartoon in grey and blue) occupies the same space as the BDM88855 (**9**) (carbon atoms, green; nitrogen atoms, blue). In the inhibitor-bound structure (marine cartoon), the Lys940 side chain adopts a conformation similar to that of the O state in the wildtype AcrB protein (see **Fig. S5**).

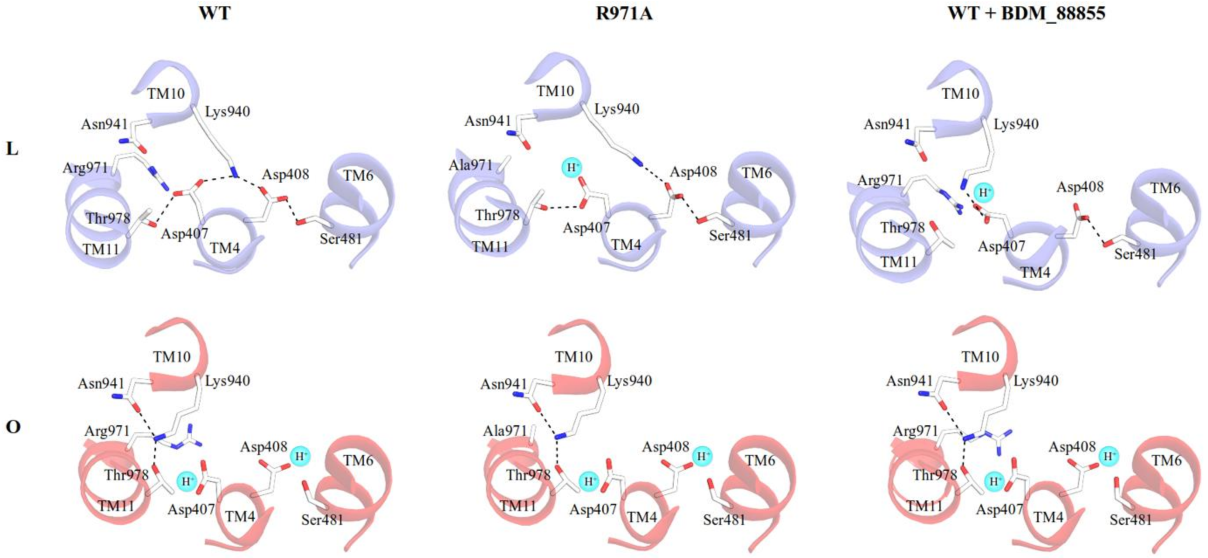

Fig. S5: Configuration of the proton-binding site in AcrB in the absence or presence of BDM88855 **(9).** The TM helices 4, 6, 10 and 11 of the L (blue cartoon) and O (red) protomers are indicated for the apo-wildtype AcrB structure (PDB: 4DX5[2]), the R971A variant (PDB: 4U96[3]) and the BDM88855 (**9**)-bound wildtype structure (this work). The side chains are shown as white sticks with oxygen atoms (red) and nitrogen atoms (blue). In light blue, the putative protonation of the D407 and D408 side chains is indicated. The protonation states of D407 and D408 in the inhibitor free structures are inferred from previous studies[2,3].

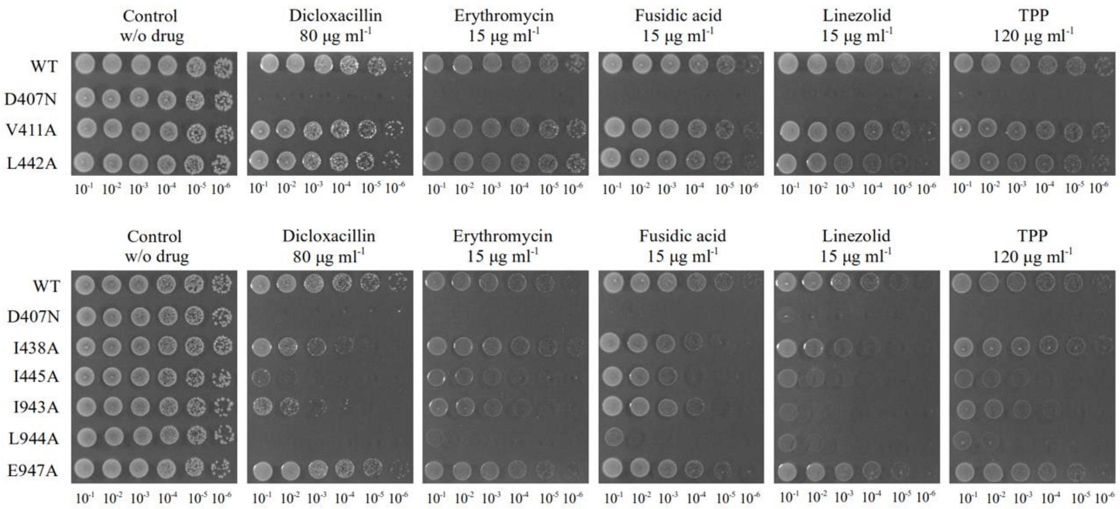

Fig. S6: Drug susceptibility profiles of *E. coli* BW25113 Δ*acrB* producing single-alanine substitution AcrB variants. Assays were performed by spotting diluted cell cultures (dilution given below the plate figures) of AcrB variants indicated at the left of the plate rows.

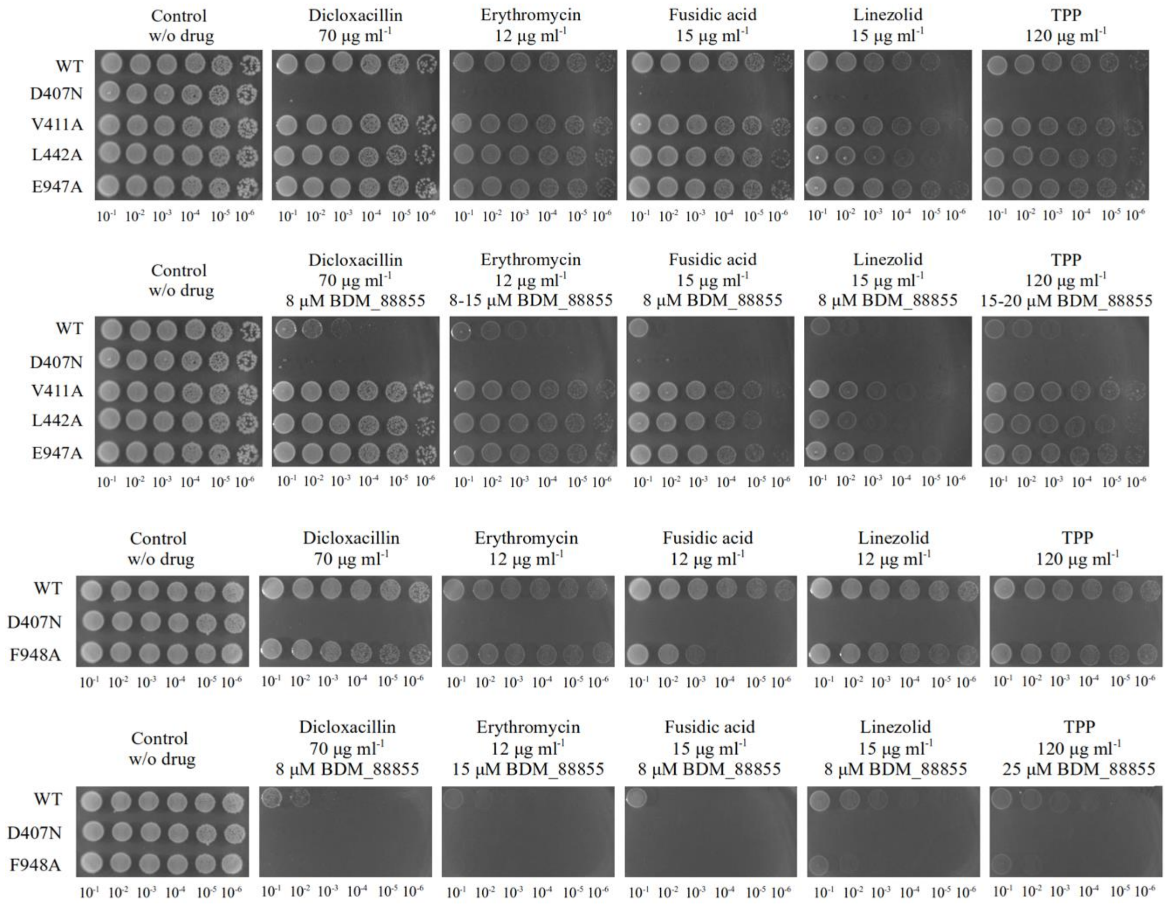

Fig. S7: Drug susceptibility profiles of *E. coli* BW25113 Δ*acrB* producing functional AcrB variants in the absence and presence of BDM88855 **(9).** Assays were performed by spotting diluted cell cultures (dilution given below the plate figures) of AcrB variants indicated at the left of the plate rows.

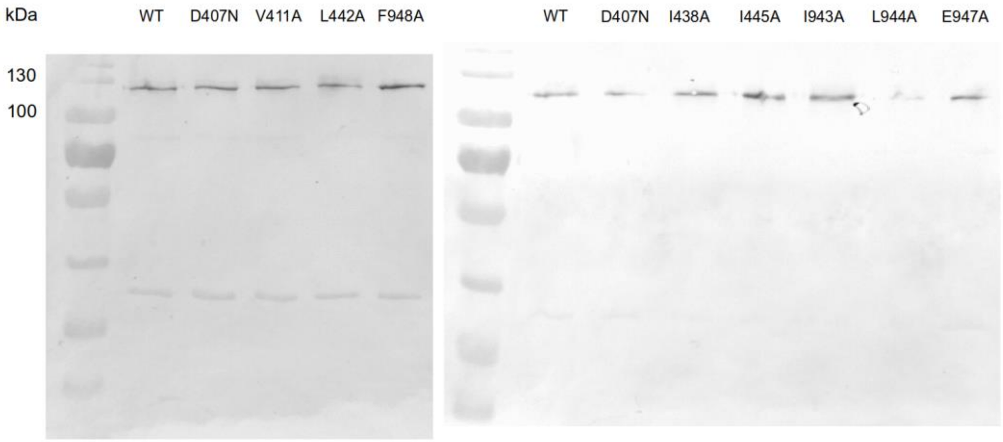

Fig S8: Western blot analysis of equal amounts of *E. coli* cell extracts producing the indicated AcrB protein. Blots were probed with anti-AcrB antibody/goat-anti-rabbit antibody coupled to alkaline phosphatase (see Methods). Wildtype and AcrB variants were expressed at similar levels, except the AcrB-L944A variant. Experiments were performed twice.

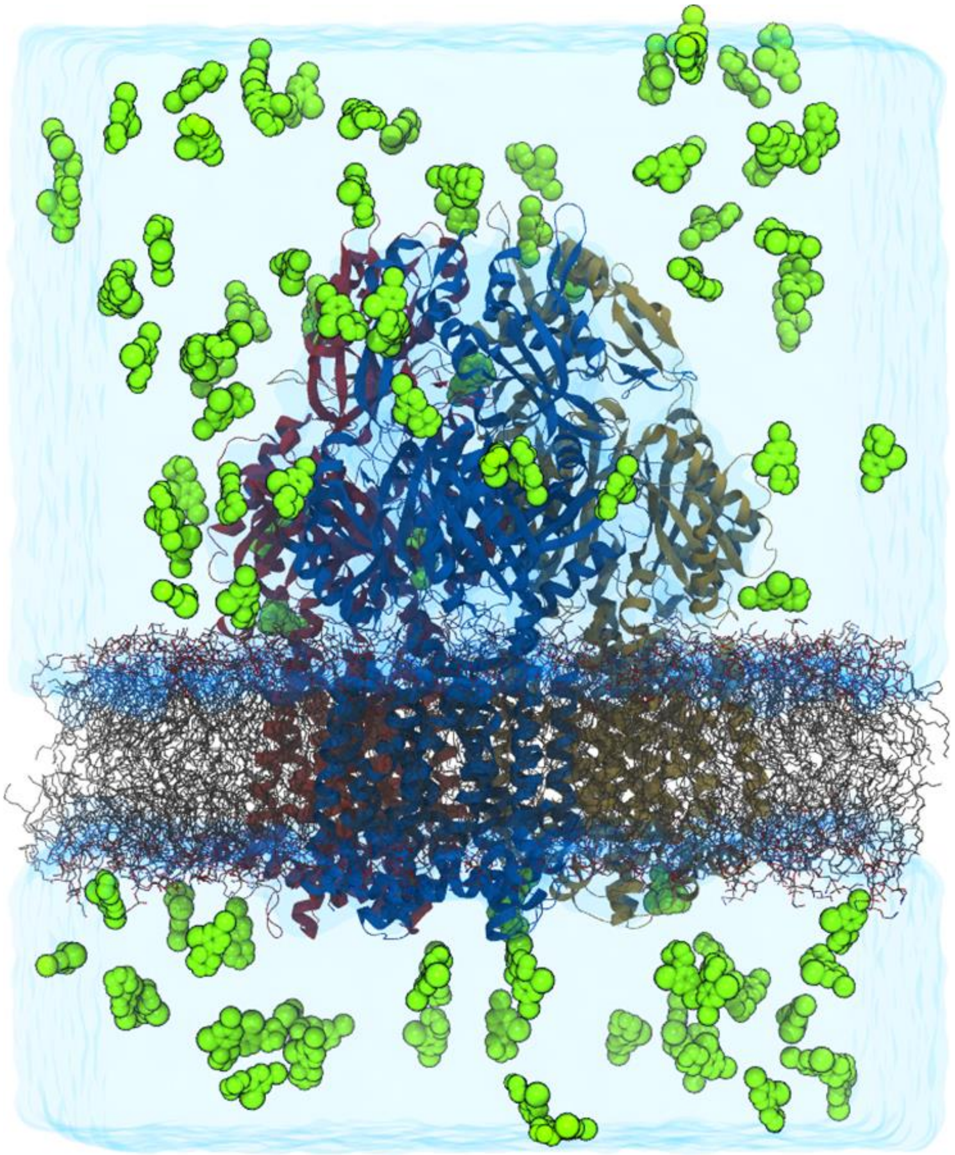

Fig. S9: Model system setup to investigate the putative uptake route of BDM88832 (**8**) towards its experimental binding site in the TM region of the L monomer of AcrB. The protein is shown in cartoon representation (L, T and O protomers in blue, dark yellow and red, respectively) embedded in a model phospholipid bilayer composed by POPE and POPG in a 2:1 ratio (thin lines colored by atom type: C, O, N in grey, red, blue respectively). 100 ligand molecules (shown by green spheres) were randomly placed within a 0.1 M NaCl water solution box (boundaries shown in light blue transparent surface) enclosing the protein and the phospholipid bilayer.

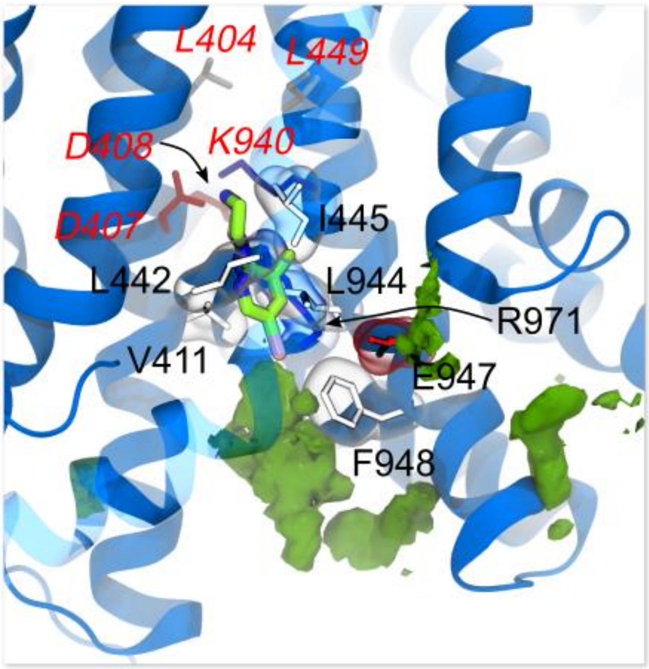

Fig. S10: Accumulation of BDM88832 (**8**) at the cytoplasmic entrance of the TM domain of the L protomer of AcrB. Density plots show the preferred locations of the inhibitor in green surfaces (cumulative results from 5 independent MD simulations of 2 μs in length each; isovalue set to 3). The L protomer of AcrB is shown in blue ribbons, with the TM helix 5 transparent for the sake of clarity. The X-ray conformation of the compound (after superposition of the TM domain of the experimental structure of AcrB to that of the closest-to-average conformation extracted from the cumulative trajectory) is shown by sticks colored by atom type (C, N, I and Cl in green, blue, pink and yellow, respectively). Residues L404, D407, D408, L449 and K940 lining the upper part of the binding site are shown by dark sticks colored by residue type (acidic: red; basic: blue; apolar: grey) and labeled red. Residues V411, L442, I445, L944, E947, F948, R971 (the latter actually being Ala in the experimental structure considered for the structural alignment), lining the bottom of the site, are also shown by sticks englobed by transparent surfaces and colored by type, and labeled black.

Supplementary Methods

General

Solvents for synthesis, analysis and purification were purchased as analytical grade from commercial suppliers and used directly without further purification. Chemical reagents were purchased from Fisher scientific, Fluorochem, Enamine or Sigma-Aldrich as reagent grade and used without further purification.

LC-MS Waters system was equipped with a 2747 sample manager, a 2695 separation module, a 2996 photodiode array detector (200-400 nm) and a Micromass ZQ2000 detector (scan 100-800). XBridge C18 column (50 mm x 4.6 mm, 3.5µm, Waters) was used. The injection volume was 20 µL. A mixture of water and acetonitrile was used as mobile phase in gradient-elution. The pH of the mobile phase was adjusted with HCOOH and NH_4OH to form a buffer solution at pH 3.8. The analysis time was 5 min (at a flow rate at 2 mL/min), 10 min (at a flow rate at 1 mL/min) or 30 min (at a flow rate at 1 mL/min). Purity (%) was determined by reversed phase HPLC, using UV detection (215 nm). All final compounds showed purity greater than 95%.

HRMS analysis was performed on a LC-MS system equipped with a LCT Premier XE mass spectrometer (Waters), using a XBridge C18 column (50 mm x 4.6 mm, 3.5µm, Waters). A gradient starting from 98% H_2O 5 mM Ammonium Formate pH 3.8 and reaching 100% MeCN 5 mM Ammonium Formate pH 3.8 within 3 min at a flow rate of 1 mL/min was used.

NMR spectra were recorded on a Bruker DRX-300 spectrometer. The results were calibrated to signals from the solvent as an internal reference [e.g. 2.50 (residual DMSO-d_6) and 39.52 (DMSO- d_6) ppm for ^{1}H and ^{13}C NMR spectra respectively]. Chemical shifts (δ) are in parts per million (ppm) downfield from tetramethylsilane (TMS). The assignments were made using one-dimensional (1D) ^{1}H and ^{13}C spectra and two-dimensional (2D) HSQC-DEPT, COSY and HMBC spectra. NMR coupling constants (J) are reported in Hertz (Hz), and splitting patterns are indicated as follows: s for singlet, brs for broad singlet, d for doublet, t for triplet, q for quartet, dd for doublet of doublet, ddd for doublet of doublet of doublet, m for multiplet, δ for chemical shift, *J* for coupling constant.

HRMS and NMR spectra for compounds BDM73185 (**1**), BDM88832 (**8**), BDM88855 (**9**) and BDM88855.HCl (**9'**) are provided as Source data file.

Flash chromatography was performed using a Puriflash PF-430 with silica gel cartridges (Buchi silica 40 µm). ELSD and UV detection (254 nm) were used to collect the desired product. Reverse flash chromatography was performed using a CombiFlash® Rf200 with C_{18} cartridges (Buchi C_{18} 40 µm). UV detection (215 and 254 nm) was used to collect the desired product.

Abbreviations

DCM, dichloromethane; DMSO, dimethylsulfoxide; EtOAc, ethyl acetate; MeCN, acetonitrile; MeOH, methanol; ovn, overnight; RT, room temperature; TEA, triethylamine.

1-[3-chloro-5-(trifluoromethyl)-2-pyridyl]piperazine (BDM73185 (1))

Compound **1** was purchased from Maybridge. White powder; Purity (215 nm) > 99%; ^{1}H NMR (300 MHz, DMSO-d_6): δ 2.80-2.83 (m, 4H), 3.34-3.38 (m, 4H), 8.14-8.16 (m, 1H), 8.52-8.54 (m, 1H); ^{13}C NMR (75 MHz, DMSO-d_6): δ 45.5, 49.6, 118.0 (q, $^2J_{CF}$ = 33 Hz), 119.7, 123.5 (q, $^1J_{CF}$ = 270 Hz), 136.1 (q, $^3J_{CF}$ = 3 Hz), 143.0 (q, $^3J_{CF}$ = 4 Hz), 159.8; HRMS (*m/z*): $[M+H]^+$ calcd. for $C_{10}H_{12}ClF_3N_3$ 266.0672; found 266.0677.

General method for the synthesis of compounds 2, 3 and 4

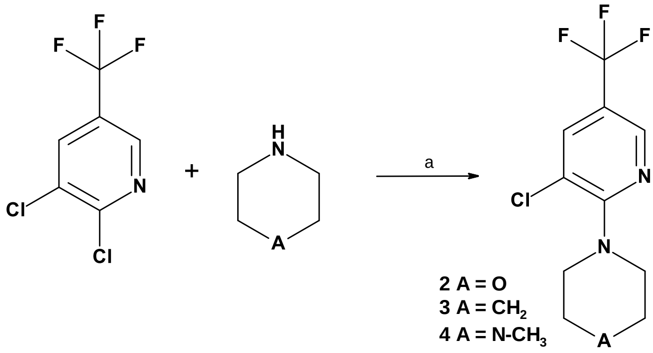

Fig. S11: Synthesis scheme of compounds **2-4**. Reagents and conditions: (a) TEA (1.3 eq.), MeCN, 50 °C, 8h-17h.

2,3-dichloro-5-(trifluoromethyl)pyridine (0.7 mmol, 1 eq), amine (1.5 eq) and TEA (1.3 eq) were dissolved in MeCN (1 mL). The mixture was heated at 50 °C for 8h to 17h, cooled to room temperature and purified by flash or reverse chromatography (Fig. S11).

4-[3-chloro-5-(trifluoromethyl)-2-pyridyl]morpholine (2)
Purification by flash chromatography (cyclohexane/AcOEt 100/0-90/10); Yield: 66% (123 mg); Colourless oil; Purity (215 nm) > 99%; ^{1}H NMR (300 MHz, CD_2Cl_2): δ 3.54 (t, *J* = 4.8 Hz, 4H), 3.85 (t, *J* = 4.8 Hz, 4H), 7.83-7.84 (m, 1H), 8.44-8.46 (m, 1H); ^{13}C NMR (75 MHz, CD_2Cl_2): δ 49.5, 67.0, 120.2 (q, $^{2}J_{CF}$ = 33 Hz), 121.2, 123.7 (q, $^{1}J_{CF}$ = 270 Hz), 136.4 (q, $^{3}J_{CF}$ = 3 Hz), 143.4 (q, $^{3}J_{CF}$ = 4 Hz), 160.2; HRMS (*m/z*): $[M+H]^+$ calcd. for $C_{10}H_{11}ClF_3N_2O$ 267.0512; found 267.0520.

3-chloro-2-(1-piperidyl)-5-(trifluoromethyl)pyridine (3)
Purification by flash chromatography (cyclohexane/AcOEt 100/0-95/5); Yield: 72% (133 mg); Colourless oil; Purity (215 nm) > 99%; ^{1}H NMR (300 MHz, CD_2Cl_2): δ 1.71-1.77 (m, 6H), 3.45-3.49 (m, 4H), 7.77-7.78 (m, 1H), 8.39-8.40 (m, 1H); ^{3}C NMR (75 MHz, CD_2Cl_2): δ 24.4, 25.8, 49.8, 118.7 (q, $^{2}J_{CF}$ = 32 Hz), 120.5, 123.5 (q, $^{1}J_{CF}$ = 270 Hz), 135.7 (q, $^{3}J_{CF}$ = 3 Hz), 142.9 (q, $^{3}J_{CF}$ = 4 Hz), 160.6; HRMS could not be determined (non-ionizable compound).

1-[3-chloro-5-(trifluoromethyl)-2-pyridyl]-4-methyl-piperazine (4)
Purification by reverse chromatography (H_2O/MeCN 90/10-0/100); Yield: 60% (117 mg); Colourless oil; Purity (215 nm) > 99%; ^{1}H NMR (300 MHz, CD_2Cl_2): δ 2.30 (s, 3H), 2.52 (t, *J* = 4.9 Hz, 4H), 3.52 (t, *J* = 4.9 Hz, 4H), 7.76-7.77 (m, 1H), 8.37-8.39 (m, 1H). ^{13}C NMR (75 MHz, CD_2Cl_2): δ 46.3, 48.9, 55.2, 119.7 (q, $^{2}J_{CF}$ = 33 Hz), 121.0, 124.0 (q, $^{1}J_{CF}$ = 271 Hz), 136.3 (q, $^{3}J_{CF}$ = 3 Hz), 143.3 (q, $^{3}J_{CF}$ = 4 Hz), 160.4; HRMS (*m/z*): $[M+H]^+$ calcd. for $C_{11}H_{14}ClF_3N_3$ 280.0828; found 280.0833.

General method for the synthesis of compounds 5 and 6

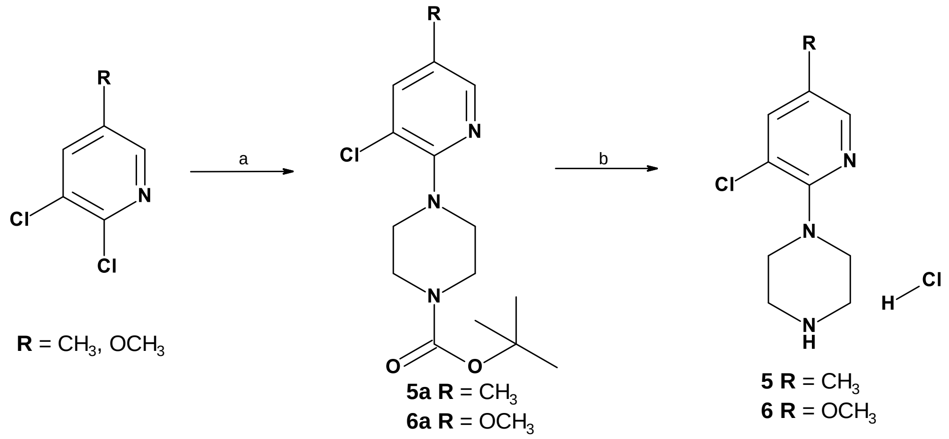

Fig. S12: Synthesis scheme of compounds **5** and **6**. Reagents and conditions: (a) Boc-piperazine (1.5 eq-2.0 eq), $Pd(OAc)_2$ (0.02 eq), BINAP (0.03 eq), *t*BuONa (1.4 eq), toluene, 110°C, ovn, (b) HCl (10 eq), 1,4-dioxane, RT, 6h-ovn.

Substituted 2,3-dichloropyridine (0.7-2.1 mmol, 1eq), Boc-piperazine (1.5-2.0 eq), $Pd(OAc)_2$ (0.02 eq), BINAP (0.03 eq) and *t*BuONa (1.4 eq) were added in a tube with dry toluene (1-3 mL). The mixture was heated at 110°C overnight, cooled to room temperature and dried under vacuum. The residue was purified by flash chromatography (cyclohexane/EtOAc 100/0-80/20) to obtain intermediates **5a** and **6a**. Each intermediate (0.15-0.41 mmol) was then dissolved in 1,4-dioxane (0.5-3 mL) and HCl 4N in 1,4-dioxane (10 eq) was added. The mixture was stirred at room temperature for 6h to overnight and then filtered and washed with petroleum ether to obtain compounds **5** and **6** (Fig. S12).

***tert*-butyl 4-(3-chloro-5-methyl-2-pyridyl)piperazine-1-carboxylate (5a)**
Yield: 58% (127 mg); White powder; LCMS (ES+) *m/z* $[M+H]^+$ 312.

***tert*-butyl 4-(3-chloro-5-methoxy-2-pyridyl)piperazine-1-carboxylate (6a)**
Yield: 55% (379 mg); Colorless oil; ^1H NMR (300 MHz, CD_2Cl_2): δ 1.45 (s, 9H), 3.09-3.15 (m, 4H), 3.51-3.57 (m, 4H), 3.80 (s, 3H), 7.28 (d, *J* = 2.7 Hz, 1H), 7.91 (d, *J* = 2.7 Hz, 1H); LCMS (ES+) *m/z* $[M+H]^+$ 328.

1-(3-chloro-5-methyl-2-pyridyl)piperazine hydrochloride (5)
Yield: 98% (99 mg); White powder; Purity (215 nm) > 99%; ^1H NMR (300 MHz, DMSO-d_6): δ 2.22 (s, 3H), 3.15-3.20 (m, 4H), 3.42 (t, *J* = 4.8 Hz, 4H), 7.73 (s, 1H), 8.09 (s, 1H), 9.21 (brs, 2H). ^{13}C NMR (75 MHz, DMSO-d_6): δ 16.6, 42.5, 45.8, 121.7, 129.2, 139.8, 145.7, 154.8; HRMS (*m/z*): $[M+H]^+$ calcd. for $C_{10}H_{15}ClN_3$ 212.0955; found 212.0963.

1-(3-chloro-5-methoxy-2-pyridyl)piperazine hydrochloride (6)
Yield: 72% (29 mg); White powder; Purity (215 nm) > 99%; ^1H NMR (300 MHz, DMSO-d_6): δ 3.14-3.22 (m, 4H), 3.30-3.36 (m, 4H), 3.80 (s, 3H), 7.61 (d, *J* = 2.7 Hz, 1H), 8.02 (d, *J* = 2.7 Hz, 1H), 9.50 (brs, 2H). ^{13}C NMR (75 MHz, DMSO-d_6): δ 42.7, 46.2, 56.4, 122.8, 125.0, 132.8, 151.0, 152.4; $[M+H]^+$ calcd. for $C_{10}H_{15}ClN_3O$ 228.0904; found 228.0903.

Method for the synthesis of compounds 7 and 8

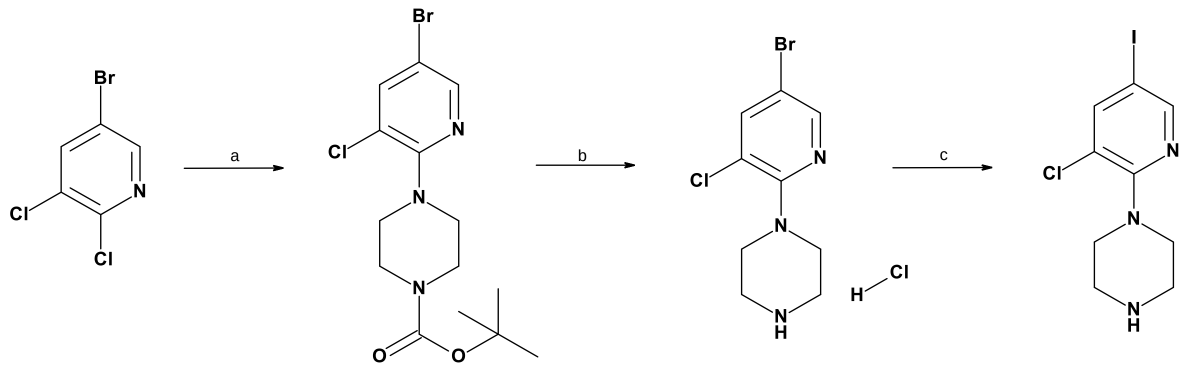

Fig. S13: Synthesis scheme of compounds **7** and **8**. Reagents and conditions: (a) Boc-piperazine (3 eq), TEA (1.3 eq), MeCN, 80°C, 2 days, (b) HCl (10 eq), 1,4-dioxane, RT, 3 days, (c) CuI (0.07 eq), NaI (2 eq), (1R,2R)-N1,N2-dimethylcyclohexane-1,2-diamine (0.1 eq), 1,4-dioxane, 110°C, 3 days.

2,3-dichloro-5-bromopyridine (0.7 mmol, 1 eq), Boc-piperazine (3 eq) and TEA (1.3 eq) were dissolved in MeCN (1 mL). The mixture was heated at 80°C for 2 days, cooled to room temperature and purified by flash chromatography (DCM/MeOH 100/0-95/5) to obtain *tert*-butyl 4-(5-bromo-3-chloro-2-pyridyl)piperazine-1-carboxylate (**7a**). Intermediate **7a** (0.59 mmol) was then dissolved in 1,4-dioxane (1 mL) and HCl 4N in 1,4-dioxane (10 eq) was added. The mixture was stirred at room temperature for 3 days and then filtered and washed with petroleum ether to obtain compound **7** (Fig. S13).

***tert*-butyl 4-(5-bromo-3-chloro-2-pyridyl)piperazine-1-carboxylate (7a)**
Yield: 85% (223 mg); White powder; LCMS (ES+) *m/z* $[M+H]^+$ 376.

1-(5-bromo-3-chloro-2-pyridyl)piperazine hydrochloride (7)
Yield: 99% (183 mg); White powder; Purity (215 nm) > 99%; ^1H NMR (300 MHz, DMSO-d_6): δ 3.15-3.20 (m, 4H), 3.47-3.52 (m, 4H), 8.18 (d, J = 2.2 Hz, 1H), 8.37 (d, J = 2.2 Hz, 1H), 9.63 (brs, 2H). ^{13}C NMR (75 MHz, DMSO-d_6): δ 42.4, 45.4, 112.4, 122.5, 141.0, 146.6, 155.9; HRMS (*m/z*): $[M+H]^+$ calcd. for $C_9H_{12}BrClN_3$ 275.9903; found 275.9902.

1-(3-chloro-5-iodo-2-pyridyl)piperazine (BDM88832 (8))
A dry 10 mL tube was charged with CuI (0.07 eq), sodium iodide (2 eq), 1-(5-bromo-3-chloro-2-pyridyl)piperazine **7** (0.096 mmol, 1 eq). The tube was purged with argon for 30 min and then 1,4-dioxane (2 mL) and (1*R*,2*R*)-N1,N2-dimethylcyclohexane-1,2-diamine (0.1 eq) were added. The mixture was heated at 110 °C for 3 days. The product was purified by flash chromatography (DCM/MeOH 100/0-90/10) and by reverse phase chromatography (H_2O/MeOH 95/5-0/100). Yield: 63% (19 mg); Pale yellow oil; Purity (215 nm) > 99%; ^1H NMR (300 MHz, CD_2Cl_2): δ 2.93-2.97 (m, 4H), 3.24-3.28 (m, 4H), 7.85 (d, J = 2.0 Hz, 1H), 8.31 (d, J = 2.0 Hz, 1H). ^{13}C NMR (75 MHz, CD_2Cl_2): δ 46.3, 50.7, 81.8, 123.4, 146.1, 151.9, 158.3; HRMS (*m/z*): $[M+H]^+$ calcd. for $C_9H_{12}ClIN_3$ 323.9765; found 323.9769

General method for the synthesis of compounds 9’ and 10

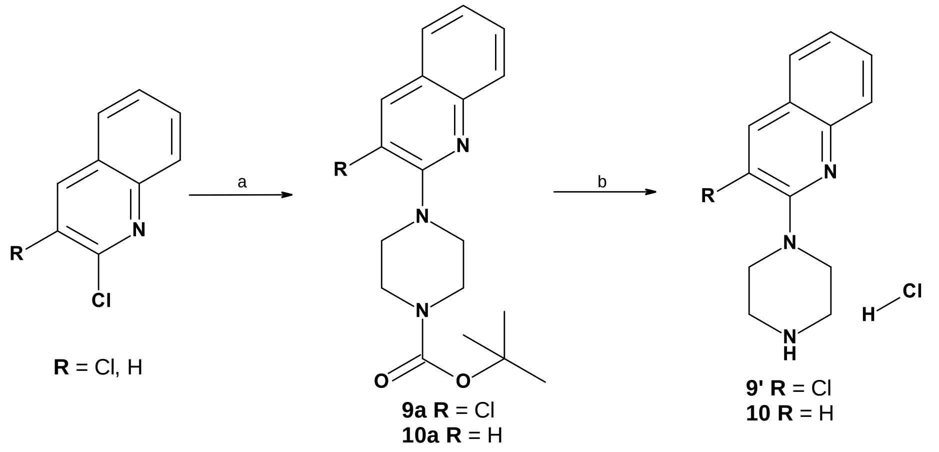

Fig. S14: Synthesis scheme of compounds **9’** and **10**. Reagents and conditions: (a) Boc-piperazine (1.5 eq), $Pd(OAc)_2$ (0.02 eq), BINAP (0.03 eq), *t*BuONa (1.4 eq), toluene, 110°C, 5h, (b) HCl (9 eq), 1,4-dioxane, RT, 4h.

Substituted quinoline (0.7-1.5 mmol, 1eq), Boc-piperazine (1.5 eq), $Pd(OAc)_2$ (0.02 eq), BINAP (0.03 eq) and *t*BuONa (1.4 eq) were added in a tube with dry toluene (1-2 mL). The mixture was heated at 110°C for 5h, cooled to room temperature and dried under vacuum. The residue was purified by flash chromatography (cyclohexane/AcOEt 100/0-90/10) to obtain intermediates **9a** and **10a**. Each intermediate (0.5-0.6 mmol, 1 eq) was then dissolved in 1,4-dioxane (1.0-1.4 mL) and HCl 4N in 1,4-dioxane (9 eq) was added. The mixture was stirred at room temperature for 4h and then filtered under vacuum and washed with petroleum ether to obtain compounds **9’** and **10** (Fig. S14).

***tert*-butyl 4-(3-chloro-2-quinolyl)piperazine-1-carboxylate (9a)**
Yield: 40% (210 mg); Beige powder; ^{1}H NMR (300 MHz, CD_2Cl_2): δ 1.47 (s, 9H), 3.40-3.45 (m, 4H), 3.59-3.63 (m, 4H), 7.40 (ddd, *J* = 1.2, 7.0, 8.1 Hz, 1H), 7.62 (ddd, *J* = 1.5, 7.0, 8.5 Hz, 1H), 7.65-7.68 (m, 1H), 7.79-7.83 (m, 1H), 8.08 (s, 1H); LCMS (ES+) *m/z* $[M+H]^+$ 348.

***tert*-butyl 4-(2-quinolyl)piperazine-1-carboxylate (10a)**
Yield: 66% (145 mg); White powder; ^{1}H NMR (300 MHz, CD_2Cl_2): δ 1.47 (s, 9H), 3.53-3.57 (m, 4H), 3.69-3.73 (m, 4H), 7.00 (d, *J* = 9.2 Hz, 1H), 7.23 (ddd, *J* = 1.3, 6.7, 8.0 Hz, 1H), 7.53 (ddd, *J* = 1.7, 6.9, 8.4 Hz, 1H), 7.61-7.67 (m, 2H), 7.93 (d, *J* = 9.1 Hz, 1H); LCMS (ES+) *m/z* $[M+H]^+$ 314.

3-chloro-2-piperazin-1-yl-quinoline hydrochloride (BDM88855.HCl (9’))
Yield: 93% (138 mg); White powder; Purity (215 nm) > 99%; ^{1}H NMR (300 MHz, DMSO-d_6): δ 3.22-3.28 (m, 4H), 3.63-3.68 (m, 4H), 7.49 (t, *J* = 7.2 Hz, 1H), 7.70 (t, *J* = 7.2 Hz, 1H), 7.80-7.89 (m, 2H), 8.49 (s, 1H), 9.71 (brs, 2H); ^{13}C NMR (75 MHz, DMSO-d_6): δ 42.4, 45.9, 121.5, 125.6, 125.7, 126.9, 127.0, 130.3, 138.3, 144.4, 155.5; HRMS (*m/z*): $[M+H]^+$ calcd. for $C_{13}H_{15}ClN_3$ 248.0955; found 248.0955.

2-piperazin-1-yl-quinoline hydrochloride (10)
Yield: 100% (116 mg); White powder; Purity (215 nm) > 99%; ^{1}H NMR (300 MHz, DMSO-d_6): δ 3.30-3.36 (m, 4H), 4.26-4.32 (m, 4H), 7.49-7.61 (m, 2H), 7.79 (t, *J* = 7.3 Hz, 1H), 7.94 (d, *J* = 7.8 Hz, 1H), 8.38-8.51 (m, 2H), 9.91 (brs, 2H); ^{13}C NMR (75 MHz, DMSO-d_6): δ 42.0, 44.0, 112.4, 119.5, 121.4, 125.1, 128.2, 132.1, 142.3, 152.5; $[M+H]^+$ calcd. for $C_{13}H_{16}N_3$ 214.1344; found 214.1349.

3-chloro-2-piperazin-1-yl-quinoline (BDM88855 (9))
2,3-dichloroquinoline (0.8 mmol, 1eq), Boc-piperazine (2 eq) and TEA (1.3 eq) were dissolved in toluene (1 mL). The mixture was heated at 110°C for 3 days, cooled to room temperature and purified

by flash chromatography (cyclohexane/AcOEt 100/0-9/1) to obtain *tert*-butyl 4-(3-chloro-2-quinolyl)piperazine-1-carboxylate (**9a**). Intermediate **9a** (0.57 mmol, 1 eq) was then dissolved in 1,4-dioxane (3 mL) and HCl 4N in 1,4-dioxane (10 eq) was added. The mixture was stirred at room temperature for 3 days and then filtered under vacuum and washed with petroleum ether. The product was purified by reverse phase chromatography (H_2O/MeOH 90/10-0/100). Yield: 14% (20 mg); Colourless oil; Purity (215 nm) 98%; ^{1}H NMR (300 MHz, DMSO-d_6): δ 3.00-3.06 (m, 4H), 3.38-3.44 (m, 4H), 7.46 (ddd, *J* = 1.2, 7.0, 8.1 Hz, 1H), 7.67 (ddd, *J* = 1.4, 6.9, 8.4 Hz, 1H), 7.78 (d, *J* = 8.4 Hz, 1H), 7.84 (dd, *J* = 1.0, 8.3 Hz, 1H), 8.44 (s,1H); ^{13}C NMR (75 MHz, DMSO-d_6): δ 44.4, 48.8, 121.7, 125.1, 125.5, 126.9, 127.0, 130.0, 137.9, 144.8, 156.3; HRMS (*m/z*): $[M+H]^+$ calcd. for $C_{13}H_{15}ClN_3$ 248.0955; found 248.0959.

Method for the synthesis of compounds **11** and **12**

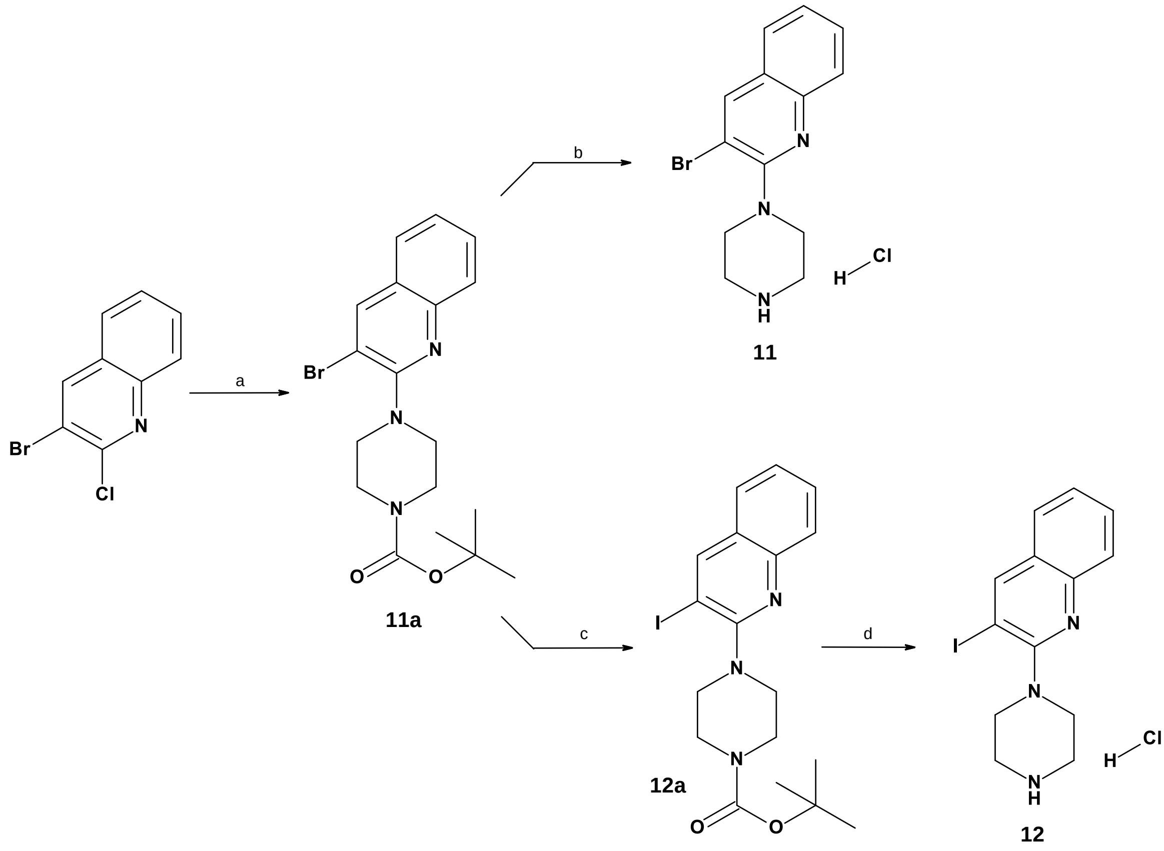

Fig. S15: Synthesis scheme of compounds **11** and **12**. Reagents and conditions: (a) Boc-piperazine (5.6 eq), TEA (1.3 eq), MeCN, 75°C (17h), (b) HCl (12.0 eq), 1,4-dioxane, 21h, (c) CuI (0.7 eq), NaI (2.0 eq), (1R,2R)-N1,N2-dimethylcyclohexane-1,2-diamine (0.3 eq), 1,4-dioxane, 110°C, 21 days, (d) HCl (16.2 eq), 1,4-dioxane, RT, 2 days.

3-bromo-2-chloro-quinoline (0.7 mmol, 1.0 eq), Boc-piperazine (1.6 eq) and TEA (1.3 eq) were dissolved in MeCN (1 mL). The mixture was heated at 75°C for 17h then Boc-piperazine (4.0 eq) was added by small portion until completed reaction. After 2 days, the reaction was cooled to room temperature and purified by flash chromatography (cyclohexane/AcOEt 100/0-90/10) to obtain *tert*-butyl 4-(3-bromo-2-quinolyl)piperazine-1-carboxylate **11a** (253 mg, Yield = 92%). Intermediate **11a** (0.63 mmol, 1.0 eq) was then dissolved in 1,4-dioxane (1.5 mL) and HCl 4N in 1,4-dioxane (9.0 eq) was added. The mixture was stirred at room temperature overnight then HCl 4N in 1,4-dioxane (3.0 eq) was added again. After 21 h, the reaction was filtered under vacuum and then washed with petroleum ether to obtain compound **11** (Fig. S15).

***tert*-butyl 4-(3-bromo-2-quinolyl)piperazine-1-carboxylate (11a)**

Yield: 92% (253 mg); Colorless oil; ^{1}H NMR (300 MHz, CD_2Cl_2): δ 1.47 (s, 9H), 3.38-3.41 (m, 4H), 3.60-3.63 (m, 4H), 7.38-7.43 (m, 1H), 7.61-7.67 (m, 2H), 7.80-7.83 (m, 1H), 8.31 (s, 1H); LCMS (ES+) *m/z* $[M+H]^+$ 392.

3-bromo-2-piperazin-1-yl-quinoline hydrochloride (11)

Yield: 100% (207 mg); Pale yellow powder; Purity (215 nm) > 99%; ^{1}H NMR (300 MHz, DMSO-d_6): 3.21-3.30 (m, 4H), 3.59-3.65 (m, 4H), 7.49 (t, *J* = 7.5 Hz, 1H), 7.71 (t, *J* = 7.5 Hz, 1H), 7.84 (dd, *J* = 7.9, 15.7 Hz, 2H), 8.69 (s, 1H), 9.70 (brs, 2H); ^{13}C NMR (75 MHz, DMSO-d_6): δ 42.5, 46.4, 111.6, 125.7, 126.3, 126.9, 127.0, 130.4, 142.1, 144.7, 156.3; HRMS (*m/z*): $[M+H]^+$ calcd. for $C_{13}H_{15}BrN_3$ 292.0449; found 292.0449.

A dry 10 mL tube was charged with *tert*-butyl 4-(3-bromo-2-quinolyl)piperazine-1-carboxylate **11a** (0.17 mmol, 1 eq), CuI (0.08 eq), sodium iodide (2.0 eq). The tube was purged with argon for 30 min then 1,4-dioxane (2.0 mL) and a solution of (1R,2R)-N1,N2-dimethylcyclohexane-1,2-diamine in 1,4-dioxane (0.1 eq) were added. The mixture was heated at 110 °C for 3 days, CuI (0.6 eq) and (1R,2R)-N1,N2-dimethylcyclohexane-1,2-diamine (0.2 eq), were added in small portions until completed reaction. After 21 days, the reaction was quenched with water, extracted twice with AcOEt. The organic layer was washed with brine and dried under vacuum. The crude product was purified by flash chromatography (cyclohexane/AcOEt 100/0-90/10) to obtain *tert*-butyl 4-(3-iodo-2-quinolyl)piperazine-1-carboxylate **12a**. Intermediate **12a** (0.05 mmol, 1.0 eq) was then dissolved in 1,4-dioxane (0.5 mL) and HCl 4N in 1,4-dioxane (10.5 eq) was added. The mixture was stirred at room temperature overnight then HCl 4N in 1,4-dioxane (5.7 eq) was added again. After 2 days, the reaction was filtered under vacuum and then washed with petroleum ether to obtain compound **12** (Fig. S15).

***tert*-butyl 4-(3-iodo-2-quinolyl)piperazine-1-carboxylate (12a)**

Yield: 30% (21 mg); Colorless oil; LCMS (ES+) *m/z* $[M+H]^+$ 440.

3-iodo-2-piperazin-1-yl-quinoline hydrochloride (12)

Yield: 79% (14 mg); Pale yellow powder; Purity (215 nm) > 99%; ^{1}H NMR (300 MHz, DMSO-d_6): δ 3.25-3.30 (m, 4H), 3.52-3.56 (m, 4H), 7.49 (ddd, *J* = 0.9, 7.0, 8.0 Hz, 1H), 7.71 (ddd, *J* = 1.2, 6.9, 8.1 Hz, 1H), 7.79-7.86 (m, 2H), 8.91 (s, 1H), 9.51 (brs, 2H); ^{13}C NMR (75 MHz, DMSO-d_6): δ 42.5, 47.1, 87.6, 125.5, 126.7, 127.2, 130.4, 145.2, 149.3, 158.7; HRMS (*m/z*): $[M+H]^+$ calcd. for $C_{13}H_{15}IN_3$ 340.0311; found 340.0311.

Supplementary References

1. Liebschner, D. *et al.* Polder maps: Improving OMIT maps by excluding bulk solvent. *Acta Crystallogr. Sect. D Struct. Biol.* **73**, 148–157 (2017).
2. Eicher, T. *et al.* Transport of drugs by the multidrug transporter AcrB involves an access and a deep binding pocket that are separated by a switch-loop. *Proc. Natl. Acad. Sci.* **109**, 5687–5692 (2012).
3. Eicher, T. *et al.* Coupling of remote alternating-access transport mechanisms for protons and substrates in the multidrug efflux pump AcrB. *Elife* **3**, e03145 (2014).
