# Supplementary material for: Pyridylpiperazine-based allosteric inhibitors of RND-type multidrug efflux pumps
Source: Nat Commun. 2022 Jan 10;13:115. doi: 10.1038/s41467-021-27726-2 (PMC8749003; doi:10.1038/s41467-021-27726-2)
Supplement: Supplementary file 3 — Reporting Summary [file 41467_2021_27726_MOESM3_ESM.pdf]

nature portfolio

Corresponding author(s): Klaas M. Pos
Marion Flipo
Ruben C. Hartkoorn

Last updated by author(s): Nov 28, 2021

Reporting Summary

Nature Portfolio wishes to improve the reproducibility of the work that we publish. This form provides structure for consistency and transparency in reporting. For further information on Nature Portfolio policies, see our Editorial Policies and the Editorial Policy Checklist.

Statistics

For all statistical analyses, confirm that the following items are present in the figure legend, table legend, main text, or Methods section.

n/a	Confirmed	
☐	☒	The exact sample size (n) for each experimental group/condition, given as a discrete number and unit of measurement
☐	☒	A statement on whether measurements were taken from distinct samples or whether the same sample was measured repeatedly
☐	☒	The statistical test(s) used AND whether they are one- or two-sided<br>*Only common tests should be described solely by name; describe more complex techniques in the Methods section.*
☒	☐	A description of all covariates tested
☒	☐	A description of any assumptions or corrections, such as tests of normality and adjustment for multiple comparisons
☐	☒	A full description of the statistical parameters including central tendency (e.g. means) or other basic estimates (e.g. regression coefficient) AND variation (e.g. standard deviation) or associated estimates of uncertainty (e.g. confidence intervals)
☐	☒	For null hypothesis testing, the test statistic (e.g. F, t, r) with confidence intervals, effect sizes, degrees of freedom and P value noted<br>*Give P values as exact values whenever suitable.*
☒	☐	For Bayesian analysis, information on the choice of priors and Markov chain Monte Carlo settings
☒	☐	For hierarchical and complex designs, identification of the appropriate level for tests and full reporting of outcomes
☒	☐	Estimates of effect sizes (e.g. Cohen's d, Pearson's r), indicating how they were calculated

Our web collection on statistics for biologists contains articles on many of the points above.

Software and code

Policy information about availability of computer code

Data collection	ImageQuant TL 8.1 for the images on drug susceptibility
Data analysis	XDS (version November 3, 2014, version BUILT=20161205, version BUILT=20161101, version March 1, 2015; version BUILT=20180808; version BUILT=20160617), Coot 0.89, Pymol 2.4.0a0, CCP4i version 7, Phenix 1.17.1, Molprobity 4.5, BUSTER 2.10.3 (Global Phasing Ltd), STARANISO (Global Phasing Ltd), ImageJ 1.52o, phenix.polder within Phenix package 1.17.1, Refmac5 within CCP4i package version 7. prinseq-lite Version 0.20.4. BWA-MEM Version 0.7.17-r1188. SAMtools Version 1.1. GATK HaplotypeCaller Version 4.2.0.0. snpEff Version 4.3

For manuscripts utilizing custom algorithms or software that are central to the research but not yet described in published literature, software must be made available to editors and reviewers. We strongly encourage code deposition in a community repository (e.g. GitHub). See the Nature Portfolio guidelines for submitting code & software for further information.

Data

Policy information about availability of data

All manuscripts must include a data availability statement. This statement should provide the following information, where applicable:

- Accession codes, unique identifiers, or web links for publicly available datasets
- A description of any restrictions on data availability
- For clinical datasets or third party data, please ensure that the statement adheres to our policy

Atomic coordinates and structure factors reported in this paper have been deposited in the Protein Data Bank under accession numbers, 7OUK (BDM88855

inhibitor bound to the transmembrane domain of AcrB) [http://doi.org/10.2210/pdb7ouk/pdb], 7OUL (BDM88832 inhibitor bound to the transmembrane domain of AcrB-R971A) [http://doi.org/10.2210/pdb7oul/pdb], 7OUM (BDM88855 inhibitor bound to the transmembrane domain of AcrB-R971A) [http://doi.org/10.2210/pdb7oum/pdb]. Atomic coordinates that were used and support the findings of this study are available in the Protein Data Bank under accession numbers 4DX5 [http://doi.org/10.2210/pdb4dx5/pdb], 4DX7 [http://doi.org/10.2210/pdb4dx7/pdb], 4U96 [http://doi.org/10.2210/pdb4u96/pdb], and 5JMN [http://doi.org/10.2210/pdb5jmn/pdb]. Whole genome sequencing data (fastq files) for parental and BDM73185 resistant E. coli isolates have been deposited at NCBI (BioProject ID: PRJNA764862)

Field-specific reporting

Please select the one below that is the best fit for your research. If you are not sure, read the appropriate sections before making your selection.

☒ Life sciences ☐ Behavioural & social sciences ☐ Ecological, evolutionary & environmental sciences

For a reference copy of the document with all sections, see nature.com/documents/nr-reporting-summary-flat.pdf

Life sciences study design

All studies must disclose on these points even when the disclosure is negative.

Sample size	Research into the initially identified efflux pump inhibitor hit were performed in singlicate to help guide subsequent research. Once more potent inhibitors were developed experiments were performed in at least 2 times (often more frequently). As the activity of the EPI was also evaluated on many antibiotic AcrB substrates, the cummulative information of these data also helps strengthen the evaluation of the EPI on AcrB. No technical replicates were included in data analysis. All the data processing and data analysis were by means of independent biological setup.<br><br>Statistical analysis was performed in Table S10, where a two-sided Student's t-test was considered appropriately robust.
Data exclusions	No samples were excluded<br>The rare exception is where errors occured due to improprer antibiotic transfer errors by the Echo Liquid Handler for the checkerboard assays. Such errors manifested themselves as wells with abundant bacterial growth while surrounding wells had no growth, which could only be explained by non-successful compound transfer.
Replication	Table 1, Data is the result of least 2 biological replicates (performed on different days)<br>Table 2, Data is the result of least 2 biological replicates (performed on different days)<br>Figure 1. Data represents the mean bacterial viability of at least 4 independent replicates (performed on different days)<br>Tables S2, S3, S4, and S5 are from single experiments. This data is generated using the original hit efflux pump inhibitor, which helped guide the research. Confirmation experiment were performed but with slightly modified conditions. Further experiments with the optimised inhibitor were performed multiple times.<br>Table S6 Data is the result of 3 biological replicates (performed on different days)<br>Table S7 Data is the result of least 2 biological replicates (performed on different days)<br>Table S10 Data is the result of least 3 biological replicates (performed on different days) and data expressed as mean ± s.e.m. In drug agar plate assay, sample size of at least N = 3-4 correspond to independent setup of biological cultures, which were defined by a setup of freshly prepared transformants, freshly picked cultures, and freshly prepared agar plate in the presence of freshly prepared antibiotics.
Randomization	In drug agar plate assay, each of the cells from independent AcrB variants are clones or transformants. In principle, colonies were picked randomly from the agar plates after transformation of plasmid into E. coli cells.
Blinding	Not applicable to all the experiments due to the need for rationale design. Negative and positive controls were included in each of the experiments

Reporting for specific materials, systems and methods

We require information from authors about some types of materials, experimental systems and methods used in many studies. Here, indicate whether each material, system or method listed is relevant to your study. If you are not sure if a list item applies to your research, read the appropriate section before selecting a response.

Materials & experimental systems

n/a	Involved in the study	
☐	☒	Antibodies
☐	☒	Eukaryotic cell lines
☒	☐	Palaeontology and archaeology
☒	☐	Animals and other organisms
☒	☐	Human research participants
☒	☐	Clinical data
☒	☐	Dual use research of concern

Methods

n/a	Involved in the study	
☒	☐	ChIP-seq
☒	☐	Flow cytometry
☒	☐	MRI-based neuroimaging

Antibodies

Antibodies used

Primary rabbit anti-AcrB antibody (dilution of 1:10,000; Neosystems, France, custom-antibody) and then, with a secondary goat anti-rabbit IgG (whole molecule)-alkaline phosphatase antibody (dilution of 1:1,500; A3687, Sigma-Aldrich, St. Louis, USA).

Validation

The commercial antibodies were verified by the manufacturers according to immunoblots and/or image on their websites (Sigma-Aldrich refers for Western Blot analysis validation to Cibelli et al., 2001 (PMID: 11298794 DOI: 10.1046/j.0953-816x.2001.01510.x). Anti-AcrB has been validated in our lab with purified AcrB as sample (e.g. Seeger MA, et al. (2008) Engineered disulfide bonds support the functional rotation mechanism of multidrug efflux pump AcrB. Nat Struct Mol Biol 15:199–205).

Eukaryotic cell lines

Policy information about cell lines

Cell line source(s)

CCL-163 from ATCC

Authentication

The cell lines used were not authenticated

Mycoplasma contamination

The cell lines were tested negative for mycoplasma contamination (kit mycoalert LONZA)

Commonly misidentified lines
(See ICLAC register)

None
