# Supplementary material for: Pyridylpiperazine-based allosteric inhibitors of RND-type multidrug efflux pumps
Source: Nat Commun. 2022 Jan 10;13:115. doi: 10.1038/s41467-021-27726-2 (PMC8749003; doi:10.1038/s41467-021-27726-2)

Control (no drug) – representative

Cells with OD_{600} of 10^{-1}–10^{-6} were pipetted from left to right.

Slide 1: Dicloxacillin (70-80 µg/ml)

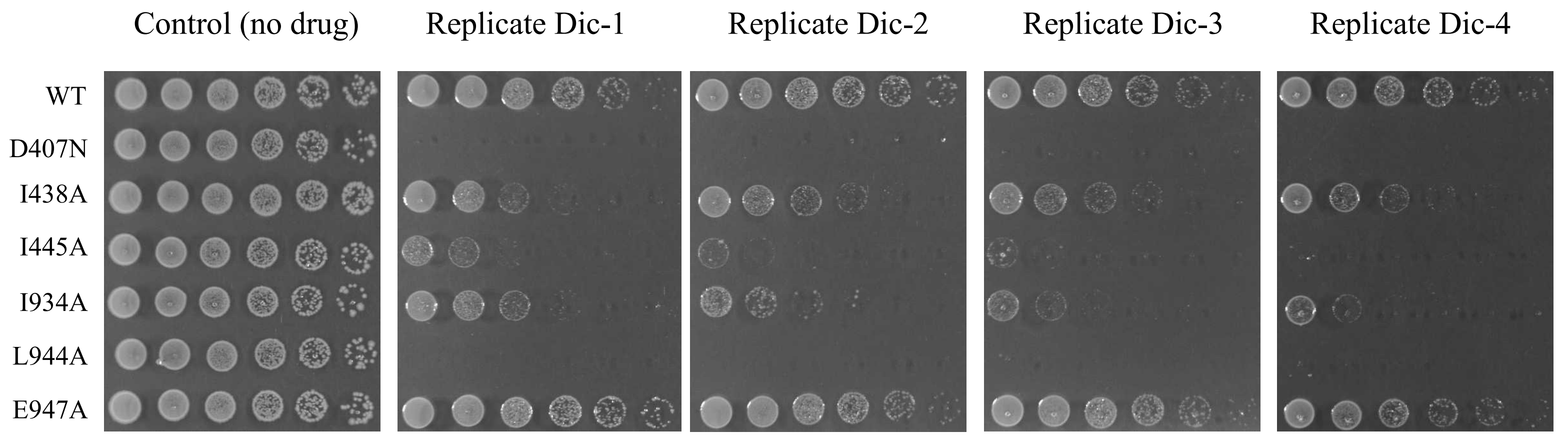

Control (no drug) – representative

Cells with OD_{600} of 10^{-1}–10^{-6} were pipetted from left to right.

Slide 2: Dicloxacillin (70-80 µg/ml)

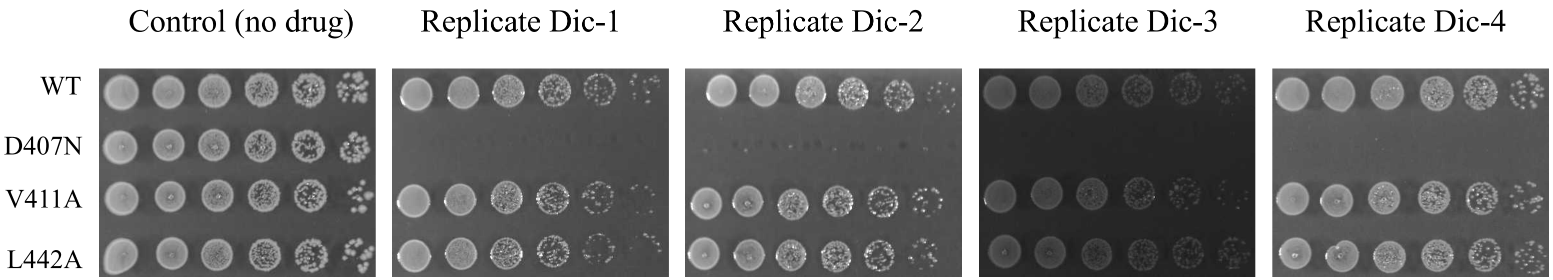

Slide 2: Dicloxacillin (80 µg/ml)

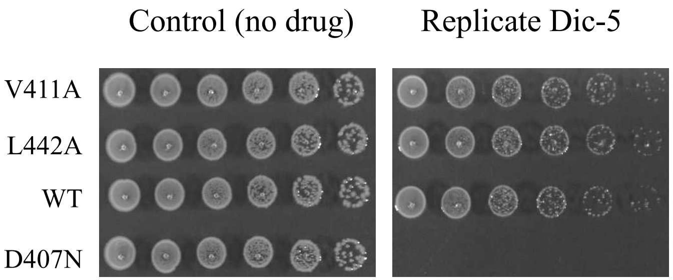

Slide 2: Dicloxacillin (70-80 µg/ml) + 8 µM BDM88855

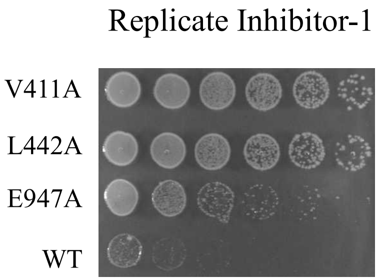

Refer ‘E947A’ to the ‘Control (no drug)’ and ‘Replicate Dic-4’ for reference.

Control (no drug) – representative

Cells with OD_{600} of 10^{-1}–10^{-6} were pipetted from left to right.

Slide 3: Dicloxacillin (80 µg/ml)

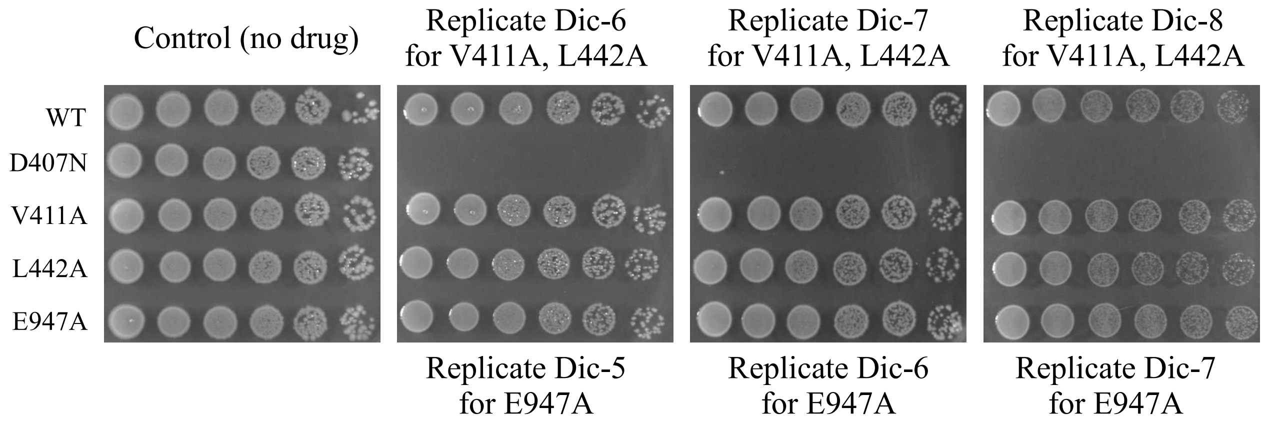

Slide 3: Dicloxacillin (70-80 µg/ml) + 8 µM BDM88855

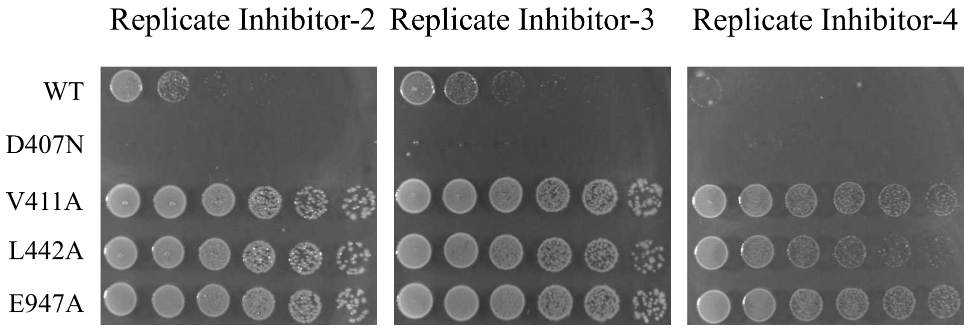

Control (no drug) – representative

Cells with OD_{600} of 10^{-1}–10^{-6} were pipetted from left to right.

Slide 4: Dicloxacillin (70-80 µg/ml)

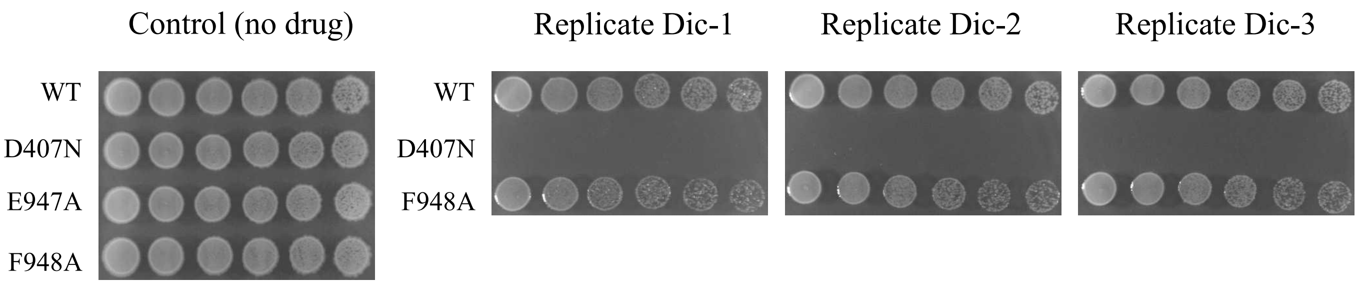

Slide 4: Dicloxacillin (70-80 µg/ml) + 8 µM BDM88855

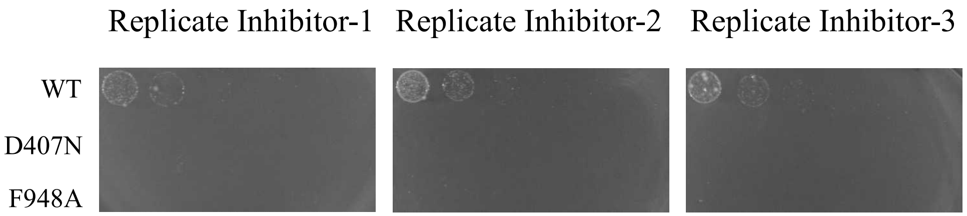

Control (no drug) – representative

Cells with OD_{600} of 10^{-1}–10^{-6} were pipetted from left to right.

Slide 5: Dicloxacillin (70-80 µg/ml)

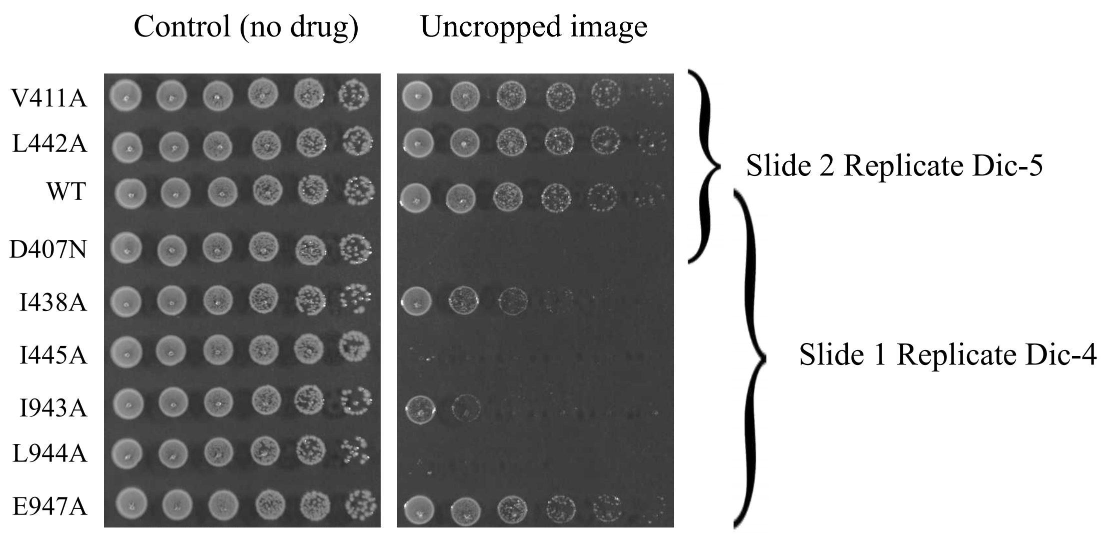

Supplement: Supplementary file 4 — Source Data [file 41467_2021_27726_MOESM4_ESM.zip › Source data Table S10 and Fig S5-S6/Source_Data_Table_S10_ and FigS5_S6_Dicloxacillin_final.pdf]
