# Supplementary material for: Pyridylpiperazine-based allosteric inhibitors of RND-type multidrug efflux pumps
Source: Nat Commun. 2022 Jan 10;13:115. doi: 10.1038/s41467-021-27726-2 (PMC8749003; doi:10.1038/s41467-021-27726-2)

Control (no drug) – representative

Cells with $OD_{600}$ of $10^{-1}$–$10^{-6}$ were pipetted from left to right.

**Slide 1: Erythromycin (15 µg/ml)**

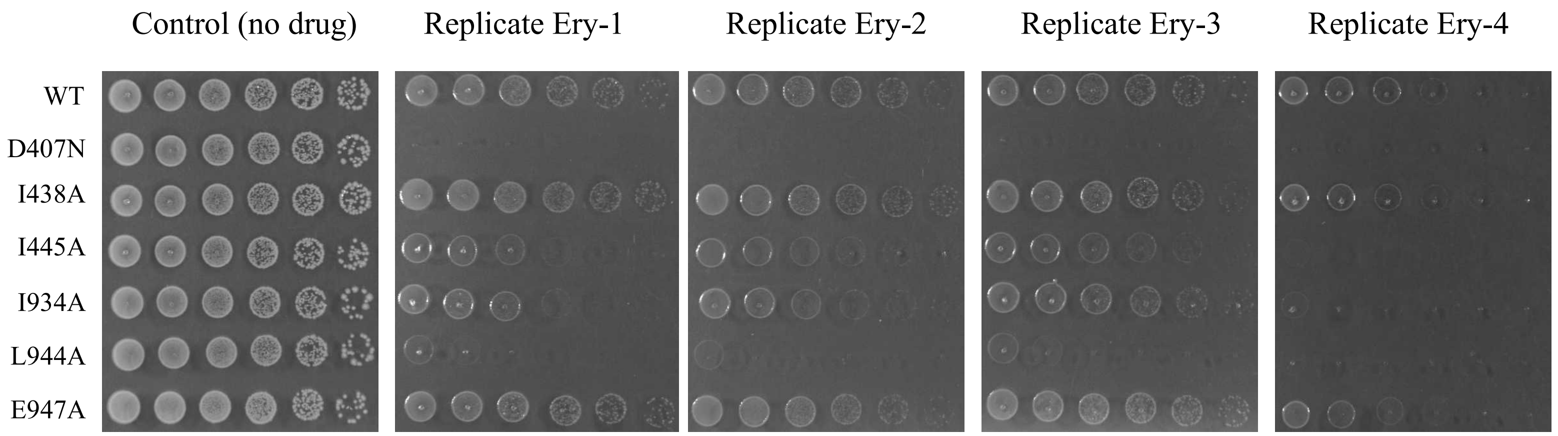

Control (no drug) – representative

Cells with $OD_{600}$ of $10^{-1}$–$10^{-6}$ were pipetted from left to right.

**Slide 2: Erythromycin (15 µg/ml)**

Control (no drug) | Replicate Ery-1 | Replicate Ery-2 | Replicate Ery-3 | Replicate Ery-4 | Replicate Ery-5

WT

D407N

V411A

L442A

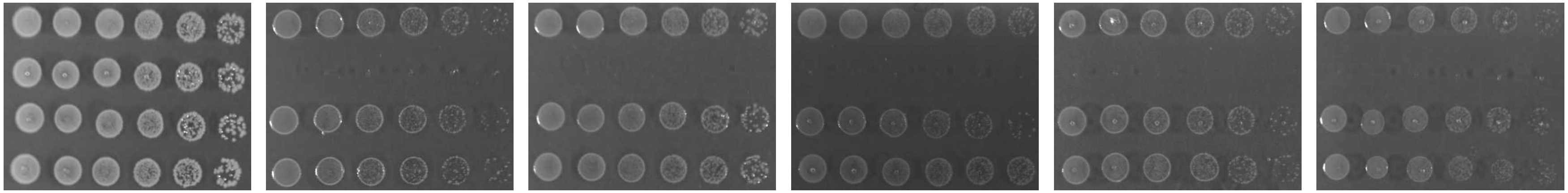

Control (no drug) – representative

Cells with $OD_{600}$ of $10^{-1}$–$10^{-6}$ were pipetted from left to right.

**Slide 3: Erythromycin (15 µg/ml)**

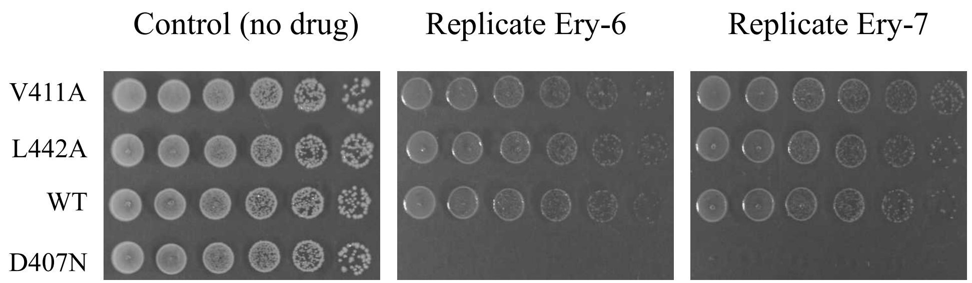


**Slide 3: Erythromycin (12-15 µg/ml) + 4-8 µM BDM88855**

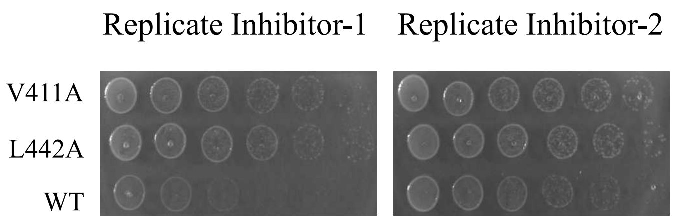

Control (no drug) – representative

Cells with $OD_{600}$ of $10^{-1}$–$10^{-6}$ were pipetted from left to right.

**Slide 4: Erythromycin (15 µg/ml)**

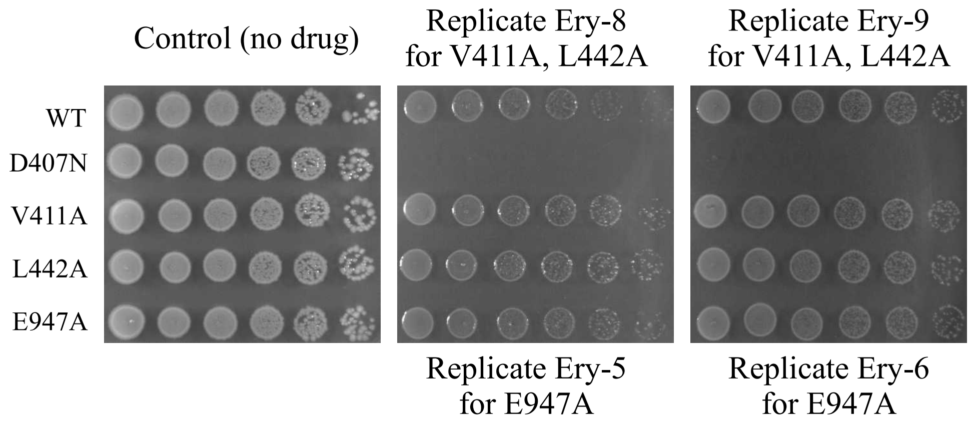


**Slide 4: Erythromycin (12-15 µg/ml) + 8 µM BDM88855**

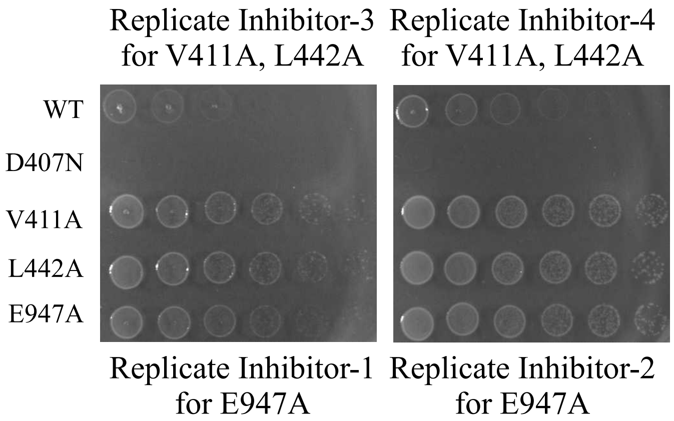

Control (no drug) – representative

Cells with $OD_{600}$ of $10^{-1}$–$10^{-6}$ were pipetted from left to right.

**Slide 5: Erythromycin (15 µg/ml)**

Control (no drug) Replicate Ery-7

WT
D407N
E947A

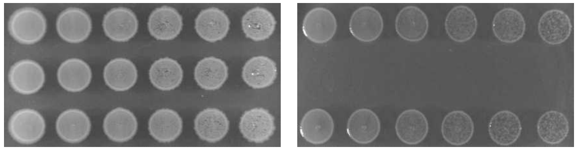

**Slide 5: Erythromycin (15 µg/ml) + 15 µM BDM88855**

Replicate Inhibitor-3

WT
D407N
E947A

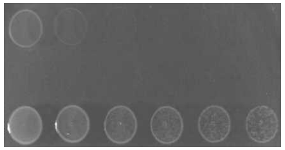

Control (no drug) – representative

Cells with $OD_{600}$ of $10^{-1}$–$10^{-6}$ were pipetted from left to right.

**Slide 6: Erythromycin (15 µg/ml)**

Control (no drug)

Replicate Ery-8 for E947A

WT

D407N

E947A

F948A

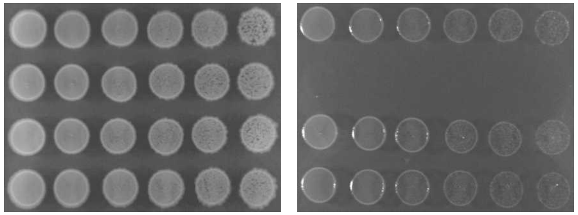

Replicate Ery-1 for F948A

**Slide 6: Erythromycin (15 µg/ml) + 15 µM BDM88855**

Control (no drug)

Replicate Inhibitor-4 for E947A

WT

D407N

E947A

F948A

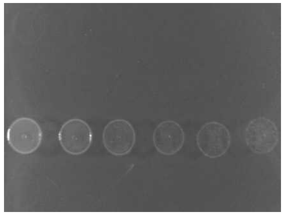

Replicate Inhibitor-1 for F948A

Control (no drug) – representative

Cells with $OD_{600}$ of $10^{-1}$–$10^{-6}$ were pipetted from left to right.

**Slide 7: Erythromycin (15 µg/ml)**

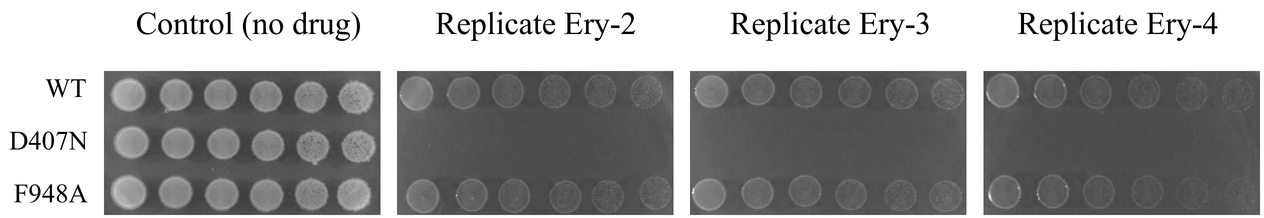


**Slide 7: Erythromycin (15 µg/ml) + 15 µM BDM88855**

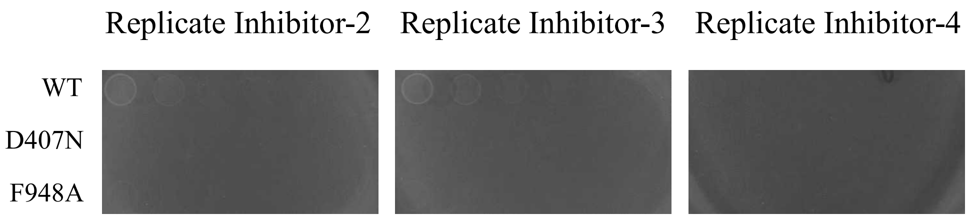

Supplement: Supplementary file 4 — Source Data [file 41467_2021_27726_MOESM4_ESM.zip › Source data Table S10 and Fig S5-S6/Source_Data_Table_S10_ and FigS5_S6_Erythromycin_final.pdf]
