# Supplementary material for: Pyridylpiperazine-based allosteric inhibitors of RND-type multidrug efflux pumps
Source: Nat Commun. 2022 Jan 10;13:115. doi: 10.1038/s41467-021-27726-2 (PMC8749003; doi:10.1038/s41467-021-27726-2)

Control (no drug) – representative

Cells with OD_{600} of 10^{-1}–10^{-6} were pipetted from left to right.

Slide 1: Fusidic acid (15 µg/ml)

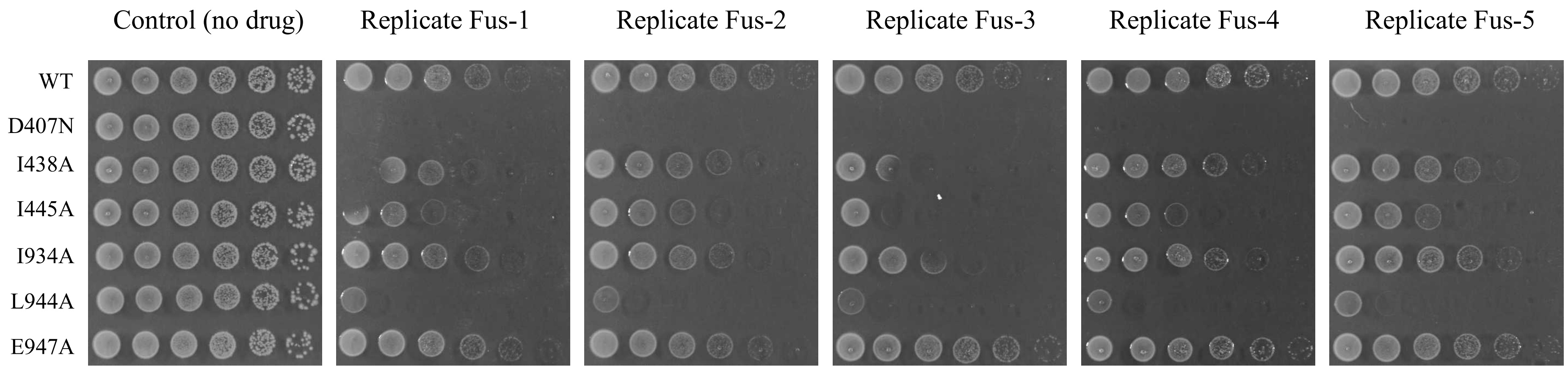

Control (no drug) – representative

Cells with OD_{600} of 10^{-1}–10^{-6} were pipetted from left to right.

Slide 2: Fusidic acid (15 µg/ml)

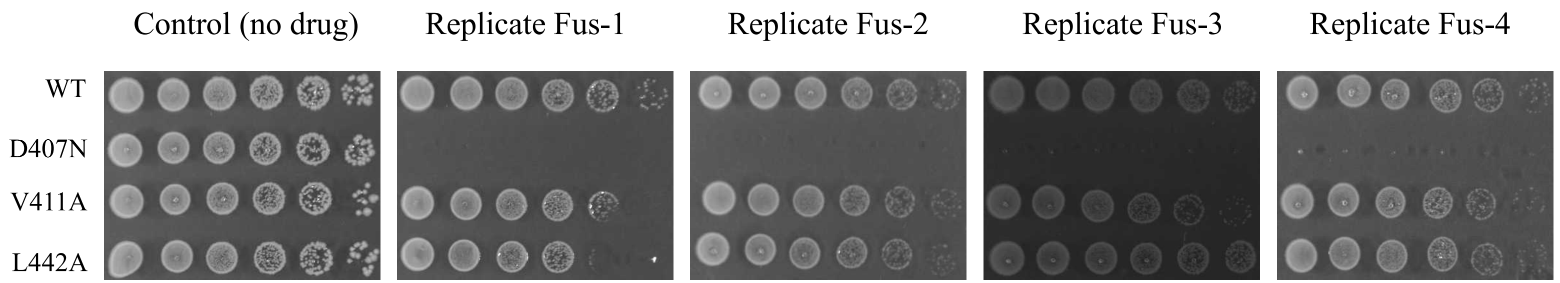

Control (no drug) – representative

Cells with OD_{600} of 10^{-1}–10^{-6} were pipetted from left to right.

Slide 3: Fusidic acid (15 µg/ml)

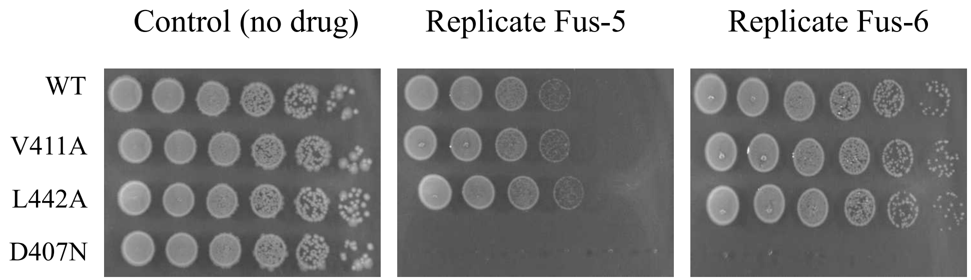

Slide 3: Fusidic acid (15 µg/ml) + 8-15 µM BDM88855

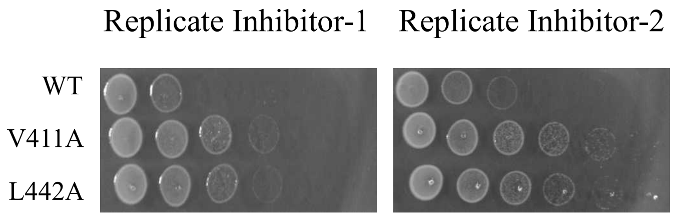

Control (no drug) – representative

Cells with OD_{600} of 10^{-1}–10^{-6} were pipetted from left to right.

Slide 4: Fusidic acid (15 µg/ml)

Control (no drug) | Replicate Fus-7 for V411A, L442A | Replicate Fus-8 for V411A, L442A | Replicate Fus-9 for V411A, L442A

WT
D407N
V411A
L442A
E947A

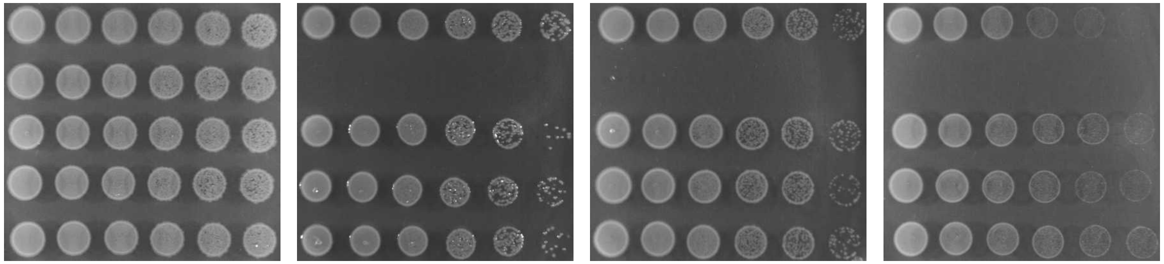

Replicate Fus-6 for E947A | Replicate Fus-7 for E947A | Replicate Fus-8 for E947A

Slide 4: Fusidic acid (15 µg/ml) + 8 µM BDM88855

Control (no drug) | Replicate Inhibitor-3 for V411A, L442A | Replicate Inhibitor-4 for V411A, L442A | Replicate Inhibitor-5 for V411A, L442A

WT
D407N
V411A
L442A
E947A

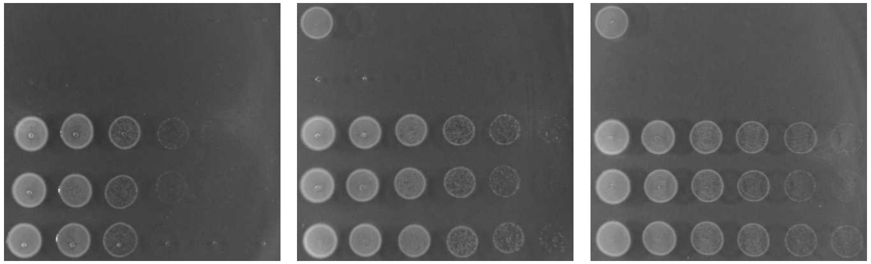

Replicate Inhibitor-1 for E947A | Replicate Inhibitor-2 for E947A | Replicate Inhibitor-3 for E947A

Slide 5: Fusidic acid (12 µg/ml)

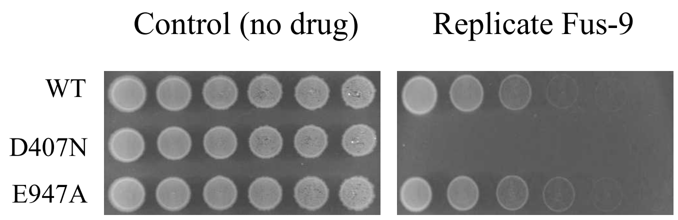

Slide 5: Fusidic acid (12 µg/ml) + 8 µM BDM88855

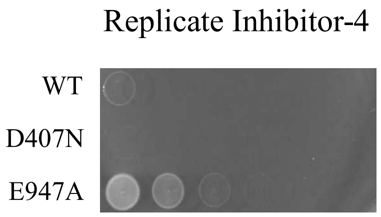

Control (no drug) – representative

Cells with OD_{600} of 10^{-1}–10^{-6} were pipetted from left to right.

Slide 6: Fusidic acid (12 µg/ml)

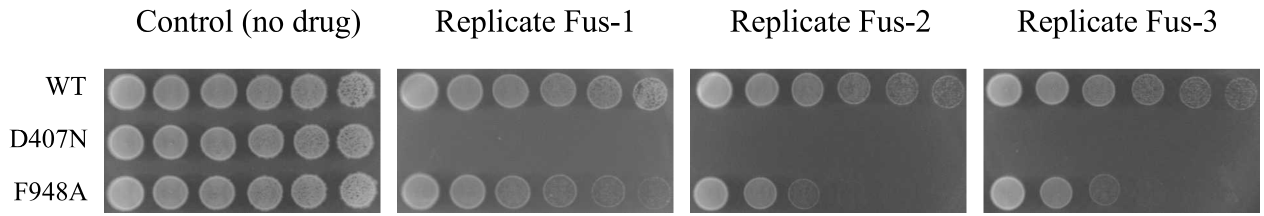

Slide 6: Fusidic acid (12 µg/ml) + 8 µM BDM88855

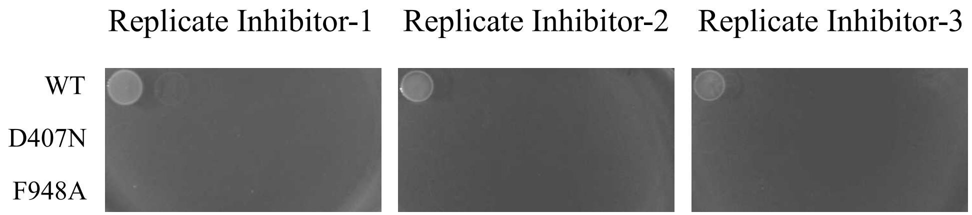

Supplement: Supplementary file 4 — Source Data [file 41467_2021_27726_MOESM4_ESM.zip › Source data Table S10 and Fig S5-S6/Source_Data_Table_S10_ and FigS5_S6_Fusidic_acid_final.pdf]
