# Supplementary material for: Pyridylpiperazine-based allosteric inhibitors of RND-type multidrug efflux pumps
Source: Nat Commun. 2022 Jan 10;13:115. doi: 10.1038/s41467-021-27726-2 (PMC8749003; doi:10.1038/s41467-021-27726-2)

Control (no drug) – representative

Cells with OD_{600} of 10^{-1}–10^{-6} were pipetted from left to right.

Slide 1: TPP (120 µg/ml)

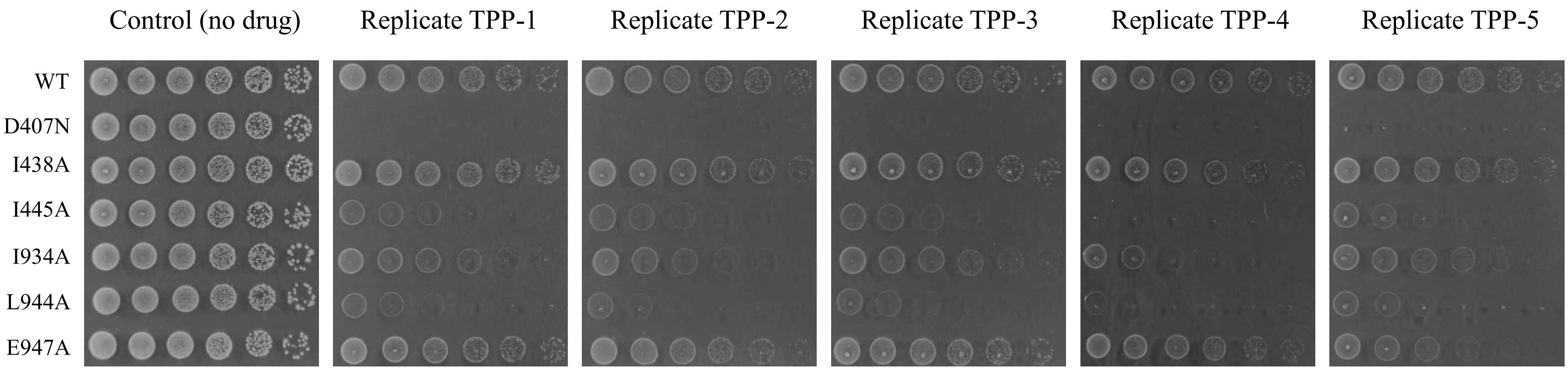

Control (no drug) – representative

Cells with OD_{600} of 10^{-1}–10^{-6} were pipetted from left to right.

Slide 2: TPP (120 µg/ml)

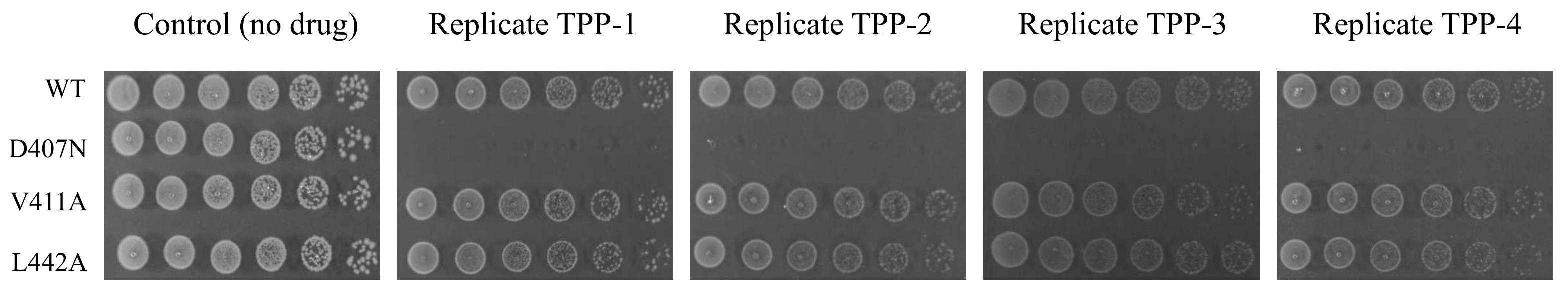

Control (no drug) – representative

Cells with OD_{600} of 10^{-1}–10^{-6} were pipetted from left to right.

Slide 3: TPP (120 µg/ml)

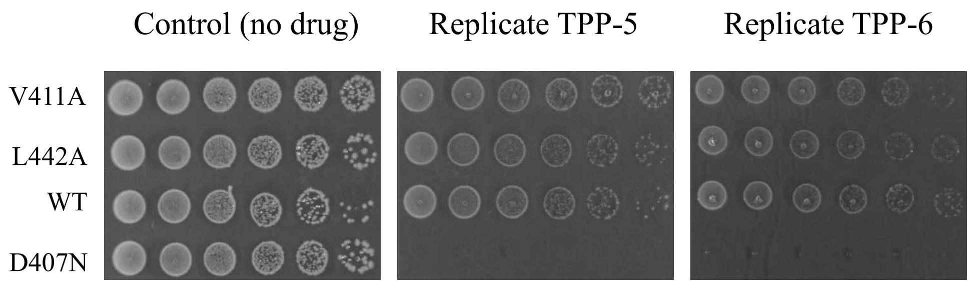

Slide 3: TPP (120 µg/ml) + 8-15 µM BDM88855

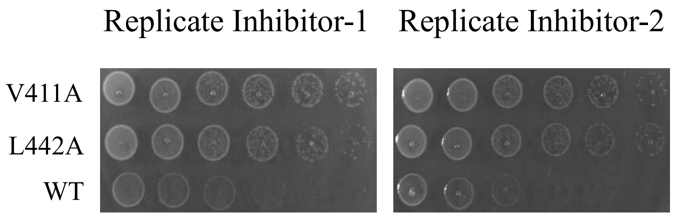

Control (no drug) – representative

Cells with OD_{600} of 10^{-1}–10^{-6} were pipetted from left to right.

Slide 4: TPP (120 µg/ml)

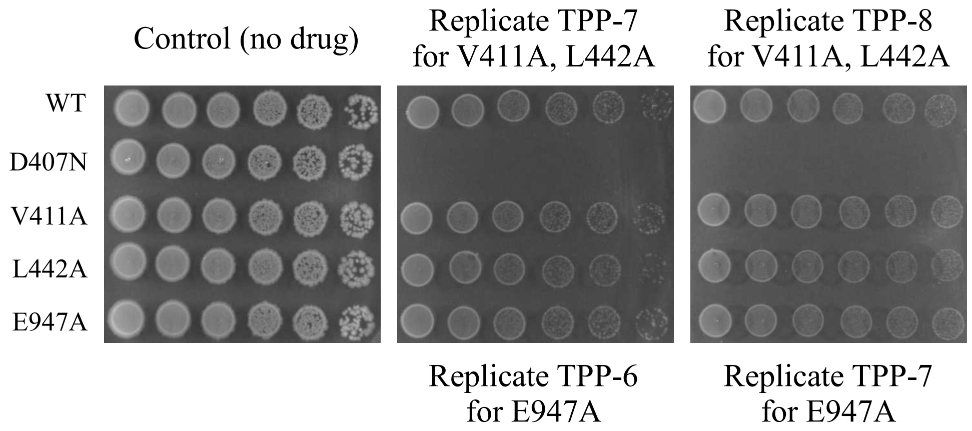

Slide 4: TPP (120 µg/ml) + 15 µM BDM88855

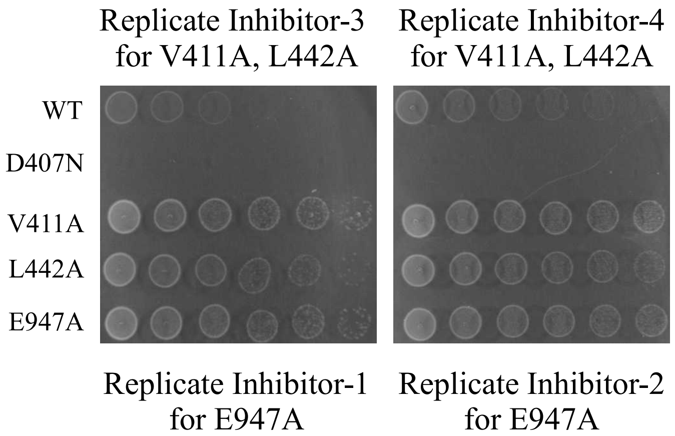

Control (no drug) – representative

Cells with OD_{600} of 10^{-1}–10^{-6} were pipetted from left to right.

Slide 5: TPP (120 µg/ml)

Control (no drug)

Replicate TPP-8
for E947A

WT
D407N
E947A
F948A

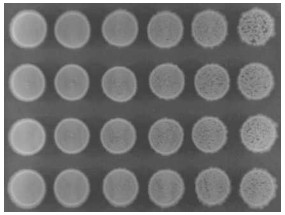

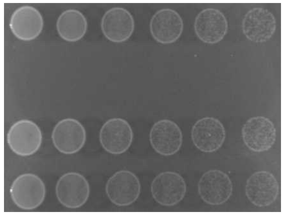

Replicate TPP-1
for F948A

Slide 5: TPP (120 µg/ml) + 20 µM BDM88855

Control (no drug)

Replicate Inhibitor-3
for E947A

WT
D407N
E947A
F948A

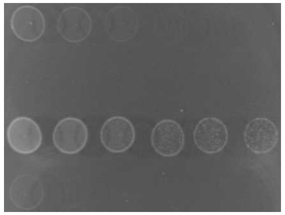

Replicate Inhibitor-1
for F948A

Refer ‘Replicate 1 for E947A’ to ‘Control (no drug)’ and ‘Replicate Lin-5’ on Slide 1.

Control (no drug) – representative

Cells with OD_{600} of 10^{-1}–10^{-6} were pipetted from left to right.

Slide 6: TPP (120 µg/ml)

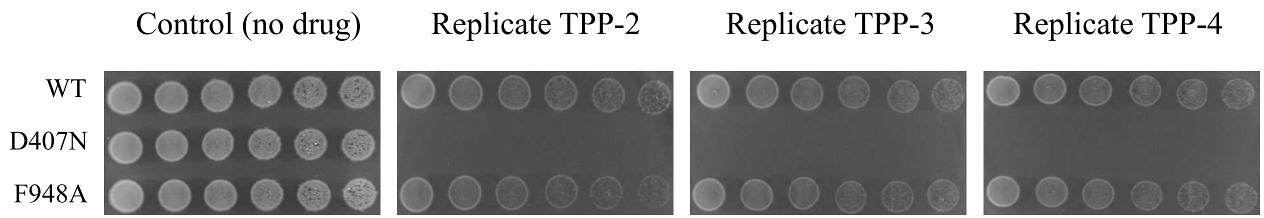

Slide 6: TPP (120 µg/ml) + 25 µM BDM88855

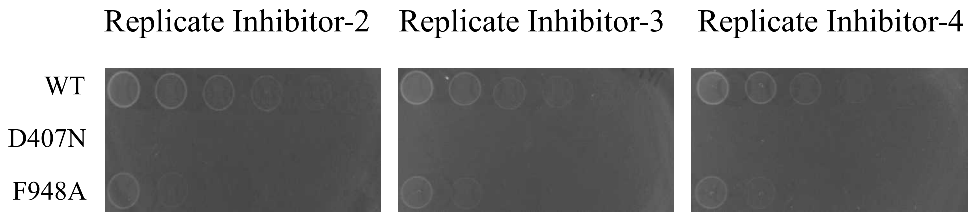

Control (no drug) – representative

Cells with OD_{600} of 10^{-1}–10^{-6} were pipetted from left to right.

Slide 7: TPP (120 µg/ml)

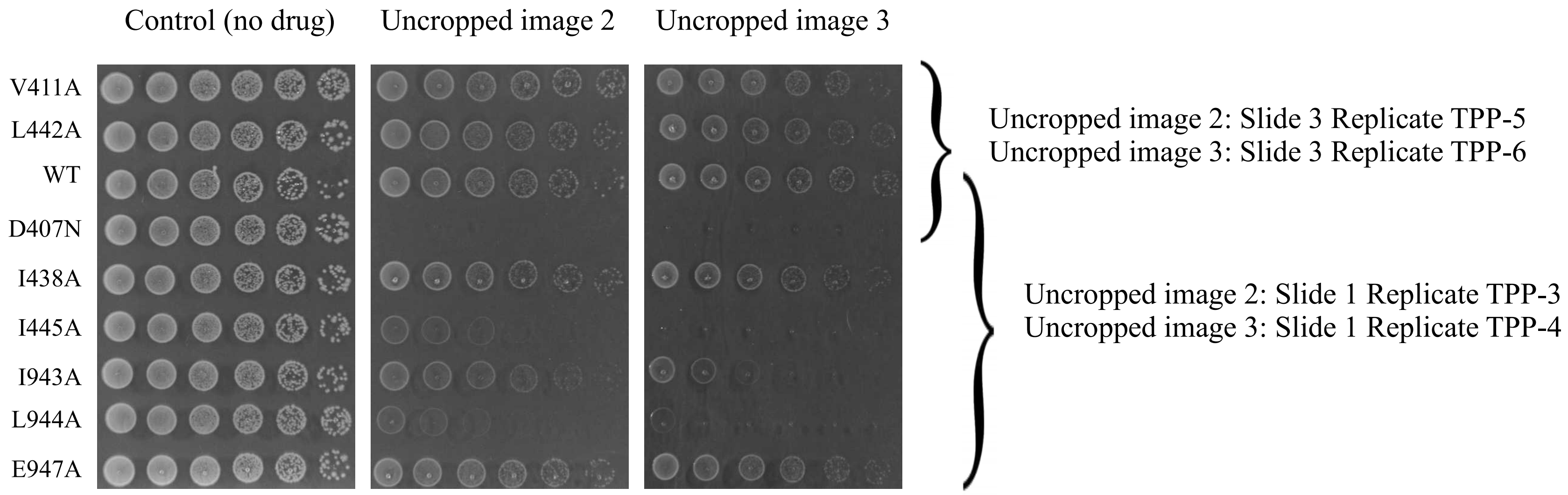

Supplement: Supplementary file 4 — Source Data [file 41467_2021_27726_MOESM4_ESM.zip › Source data Table S10 and Fig S5-S6/Source_Data_Table_S10_ and FigS5_S6_TPP_final.pdf]
