# Supplementary material for: Pyridylpiperazine-based allosteric inhibitors of RND-type multidrug efflux pumps
Source: Nat Commun. 2022 Jan 10;13:115. doi: 10.1038/s41467-021-27726-2 (PMC8749003; doi:10.1038/s41467-021-27726-2)

HRMS and NMR spectra for compounds BDM73185 (1), BDM88832 (8), BDM88855 (9) and BDM88855.HCl (9')

HRMS and NMR spectra for BDM73185 (**1**)

Elemental Composition Report

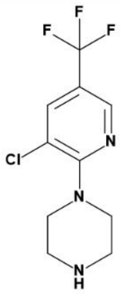

1

Single Mass Analysis
Tolerance = 10.0 PPM / DBE: min = -1.5, max = 500.0
Element prediction: Off
Number of isotope peaks used for i-FIT = 2

Monoisotopic Mass, Even Electron Ions
6467 formula(e) evaluated with 47 results within limits (up to 10 best isotopic matches for each mass)
Elements Used:
C: 0-40 H: 0-50 N: 0-12 O: 0-10 S: 0-10 Cl: 0-1 F: 0-5

SEW04036DA 104 (2.022) Cm (103:104-(94:99+108:114))

1: TOF MS ES+
2.16e+004

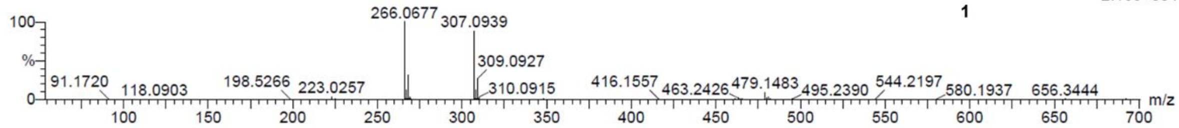

Minimum:				-1.5		
Maximum:		100.0	10.0	500.0		
Mass	Calc. Mass	mDa	PPM	DBE	i-FIT	Formula
266.0677	266.0672	0.5	1.9	4.5	0.5	C10 H12 N3 Cl F3
	266.0669	0.8	3.0	8.5	1.4	C8 H9 N9 Cl
	266.0662	1.5	5.6	3.5	2.2	C10 H14 N O3 S F2
	266.0689	-1.2	-4.5	8.5	5.2	C10 H9 N5 O3 F
	266.0687	-1.0	-3.8	4.5	5.8	C8 H11 N5 S F3
	266.0685	-0.8	-3.0	8.5	6.8	C6 H8 N11 S
	266.0685	-0.8	-3.0	2.5	6.9	C10 H17 N O2 S2 F
	266.0698	-2.1	-7.9	2.5	9.5	C9 H16 N O6 S
	266.0660	1.7	6.4	-0.5	18.6	C8 H16 N S2 F4
	266.0676	0.1	0.4	3.5	21.4	C9 H13 N O7 F

^{1}H NMR (300 MHz, DMSO-d_6)

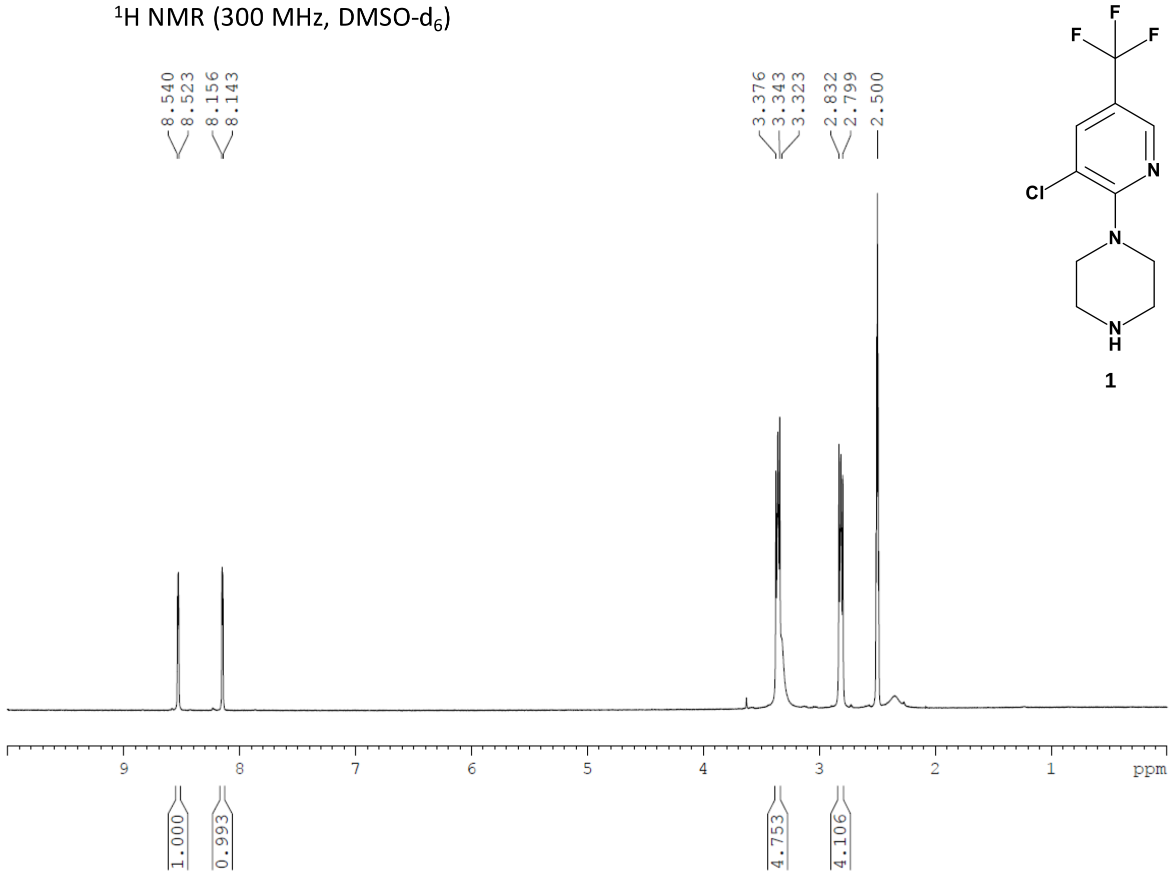

1

^{13}C NMR (75 MHz, DMSO-d_6)

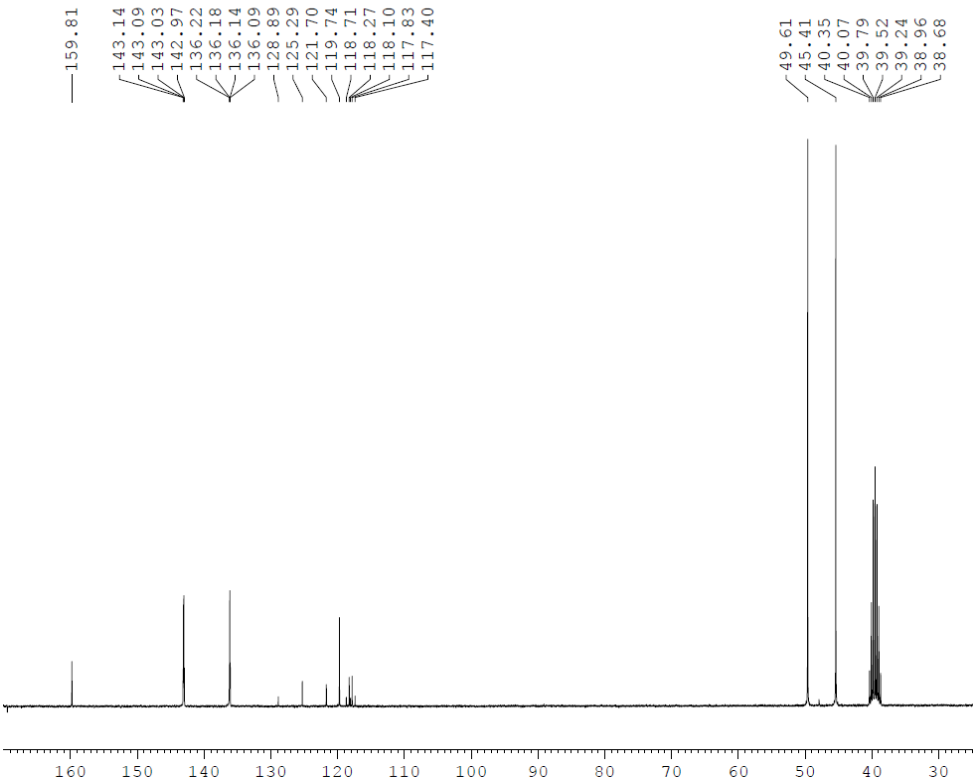

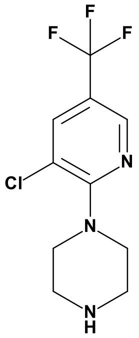

1

HRMS and NMR spectra for BDM88832 (**8**)

Elemental Composition Report

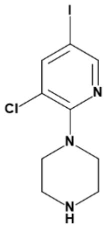

8

Single Mass Analysis

Tolerance = 100.0 mDa / DBE: min = -1.5, max = 500.0

Element prediction: Off

Number of isotope peaks used for i-FIT = 3

Monoisotopic Mass, Even Electron Ions

4817 formula(e) evaluated with 611 results within limits (up to 5 best isotopic matches for each mass)

Elements Used:

C: 9-40 H: 0-40 N: 0-10 O: 0-10 F: 0-3 Cl: 0-2 Br: 0-1 I: 0-1

PMO19HG5545 bis 102 (1.987) Cm (102:103-(96:99+104:109))

1: TOF MS ES+
1.13e+004

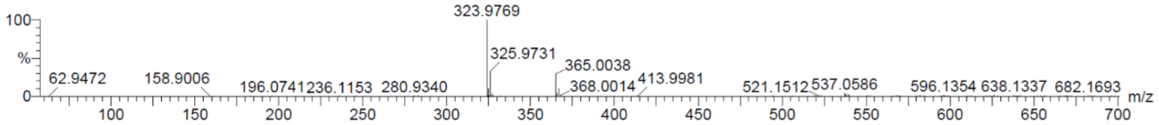

Minimum:				-1.5		
Maximum:		100.0	5.0	500.0		
Mass	Calc. Mass	mDa	PPM	DBE	i-FIT	Formula
323.9769	323.9765	0.4	1.2	4.5	7.9	C9 H12 N3 Cl I
	323.9835	-6.6	-20.4	7.5	31.4	C9 H5 N3 O6 F2 Cl
	323.9758	1.1	3.4	6.5	7.7	C9 H7 N O10 Cl
	323.9723	4.6	14.2	7.5	23.9	C10 H5 N O7 F2 Cl
	323.9772	-0.3	-0.9	11.5	48.4	C10 H3 N5 O6 Cl

^{1}H NMR (300 MHz, DMSO-d_6)

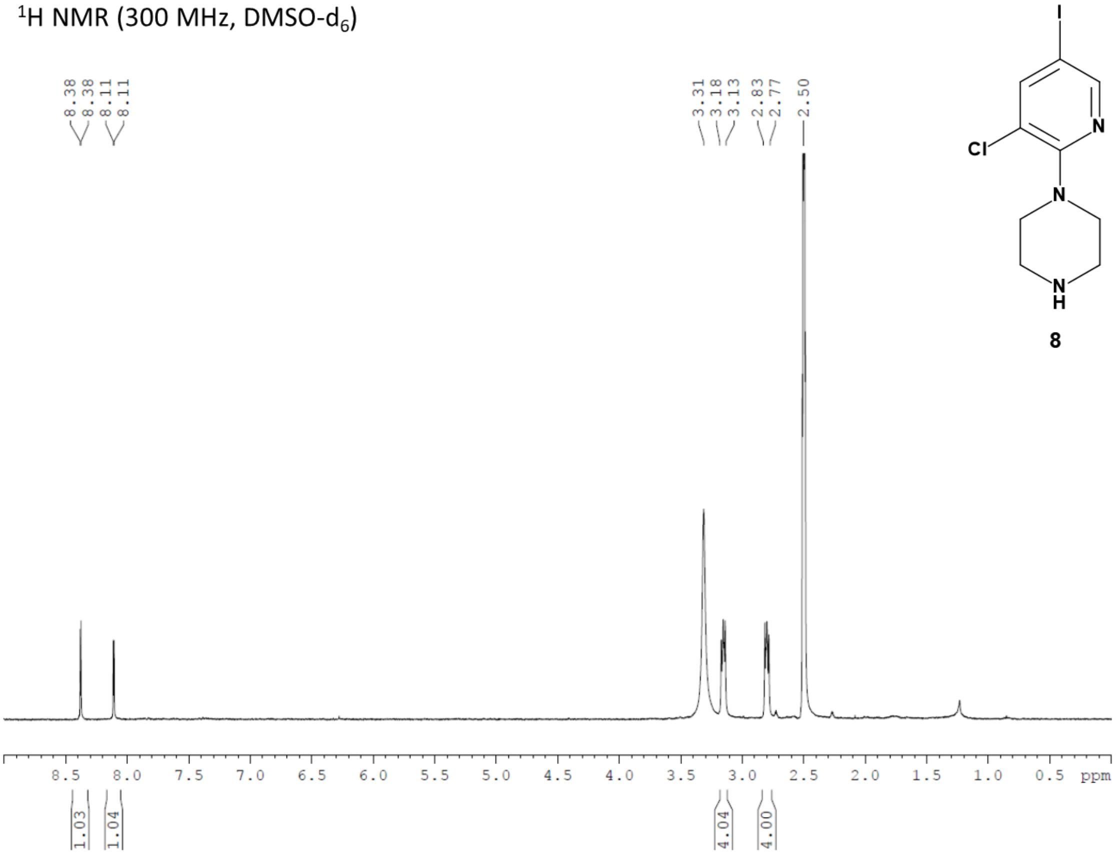

8

^{13}C NMR (75 MHz, DMSO-d_6)

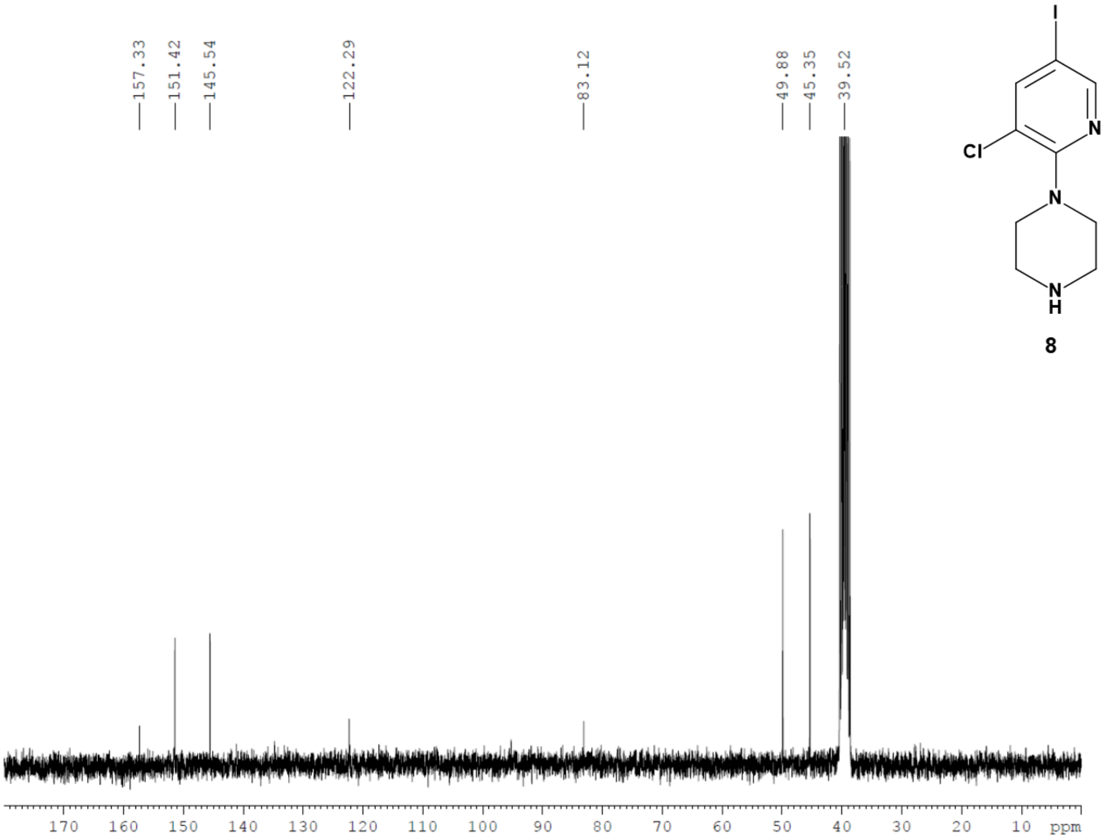

8

HRMS and NMR spectra for BDM88855 (**9**)

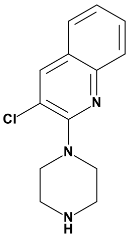

9

Elemental Composition Report

Single Mass Analysis

Tolerance = 10.0 PPM / DBE: min = -1.5, max = 500.0
Element prediction: Off
Number of isotope peaks used for i-FIT = 3

Monoisotopic Mass, Even Electron Ions
835 formula(e) evaluated with 5 results within limits (up to 5 closest results for each mass)
Elements Used:
C: 0-65 H: 0-100 N: 0-10 O: 0-16 Cl: 0-5

PMO19HG5551 BDM_88855 5uM 103 (2.005) Cm (103-(96:100+108:114))

1: TOF MS ES+
1.26e+004

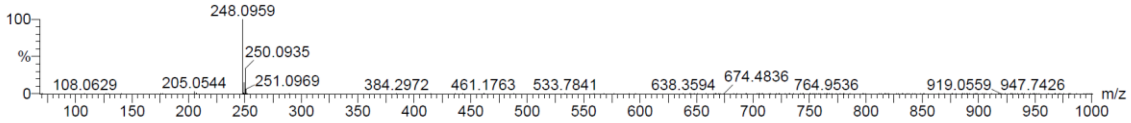

Minimum:				-1.5		
Maximum:		100.0	10.0	500.0		
Mass	Calc. Mass	mDa	PPM	DBE	i-FIT	Formula
248.0959	248.0955	0.4	1.6	7.5	4.9	C13 H15 N3 Cl
	248.0955	0.4	1.6	-0.5	2294.4	C2 H14 N7 O7
	248.0973	-1.4	-5.6	2.5	926.8	C12 H20 N Cl2
	248.0982	-2.3	-9.3	-1.5	2065.3	C6 H18 N O9
	248.0936	2.3	9.3	12.5	1960.4	C14 H10 N5

^{1}H NMR (300 MHz, DMSO-d_6)

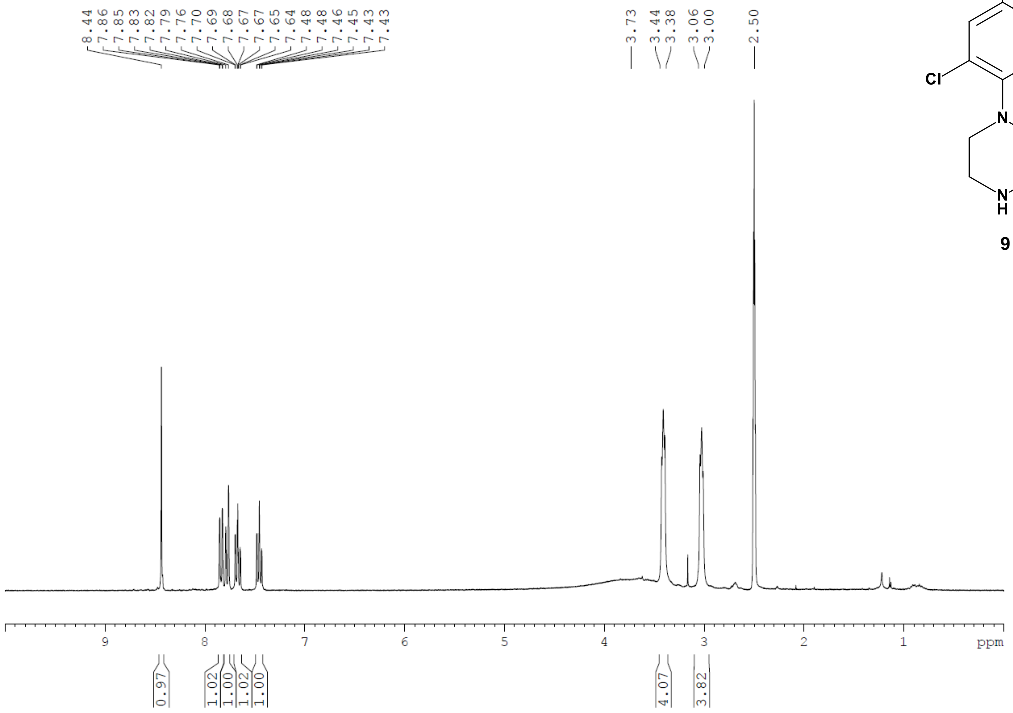

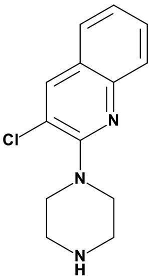

9

^{13}C NMR (75 MHz, DMSO-d$_6$)

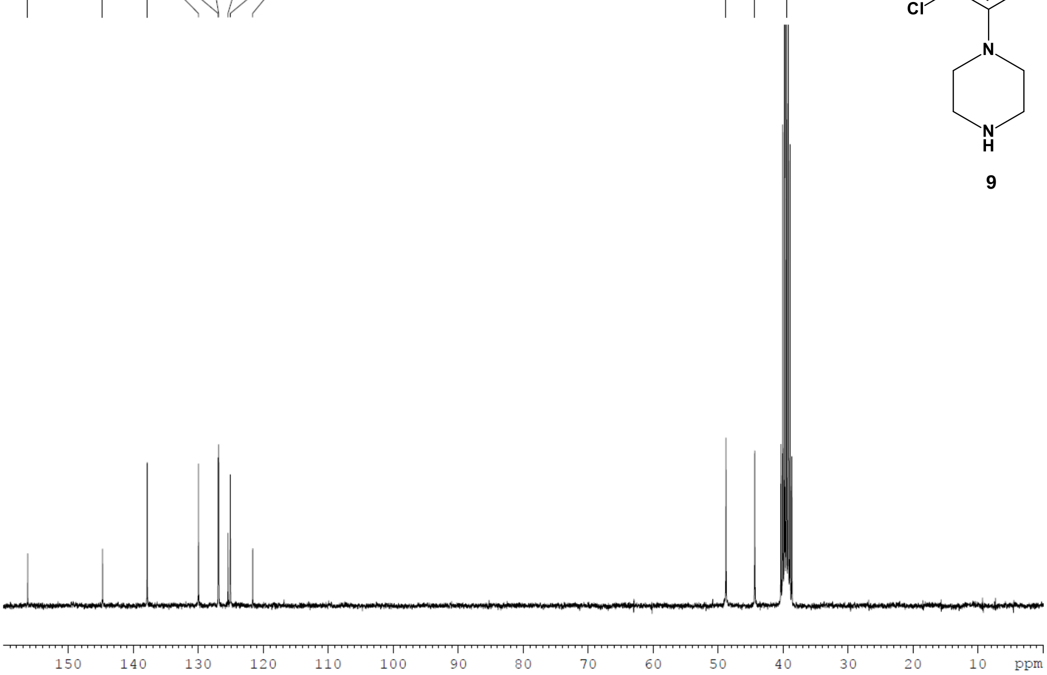

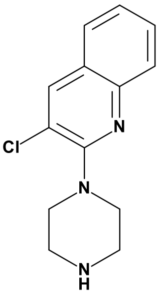

9

HRMS and NMR spectra for BDM88855.HCl (9')

Elemental Composition Report

Single Mass Analysis
Tolerance = 100.0 mDa / DBE: min = -1.5, max = 500.0
Element prediction: Off
Number of isotope peaks used for i-FIT = 3

Monoisotopic Mass, Even Electron Ions
2569 formula(e) evaluated with 529 results within limits (up to 5 best isotopic matches for each mass)
Elements Used:
C: 0-40 H: 0-40 N: 0-10 O: 0-10 F: 0-3 Cl: 0-2 Br: 0-1

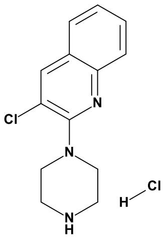

9'

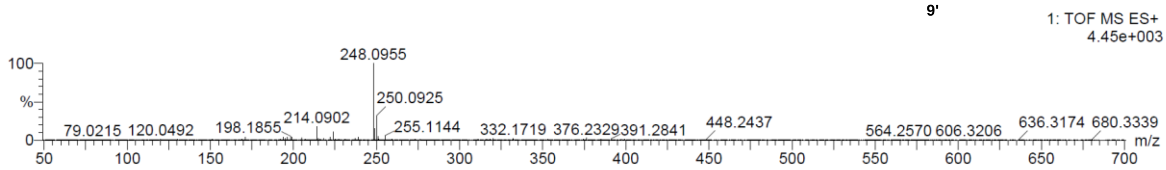

Minimum:				-1.5		
Maximum:		100.0	5.0	500.0		
Mass	Calc. Mass	mDa	PPM	DBE	i-FIT	Formula
248.0955	248.0955	0.0	0.0	7.5	1.7	C13 H15 N3 Cl
	248.0966	-1.1	-4.4	3.5	9.2	C10 H16 N3 O F Cl
	248.1018	-6.3	-25.4	3.5	13.7	C12 H17 N F2 Cl
	248.0914	4.1	16.5	3.5	27.0	C8 H15 N5 O2 Cl
	248.1053	-9.8	-39.5	2.5	35.3	C11 H19 N O3 Cl

^1H NMR (300 MHz, DMSO-d_6)

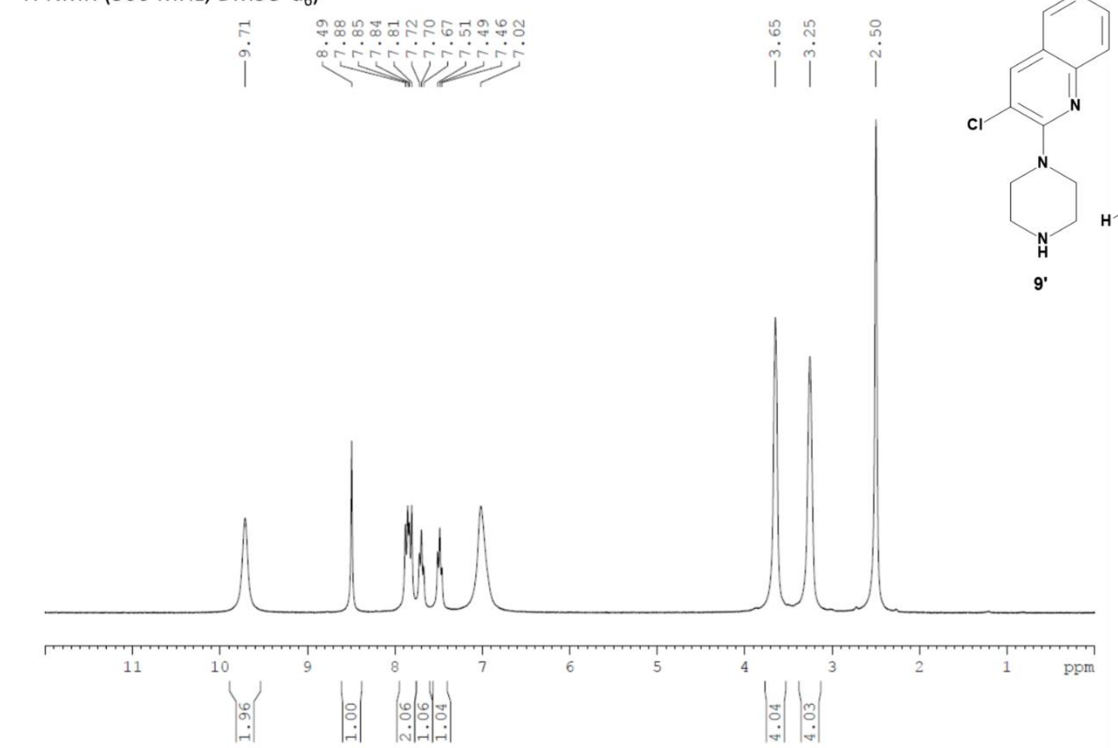

^{13}C NMR (75 MHz, DMSO-d_6)

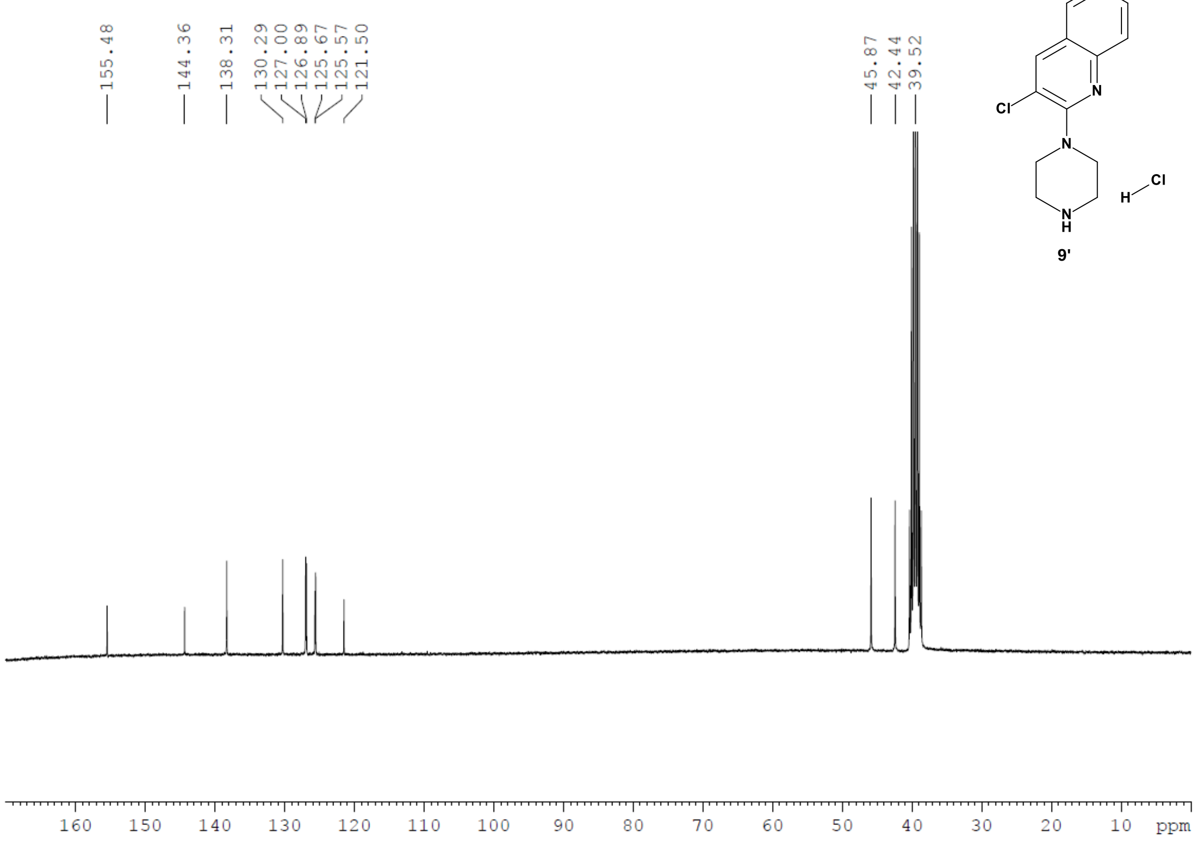

Supplement: Supplementary file 4 — Source Data [file 41467_2021_27726_MOESM4_ESM.zip › Source data chemistry HRMS NMR.pdf]
